# R-loops and impaired autophagy trigger cGAS-dependent inflammation via micronuclei formation in Senataxin-deficient cells

**DOI:** 10.1007/s00018-024-05380-3

**Published:** 2024-08-09

**Authors:** Laura Zannini, Miriana Cardano, Giordano Liberi, Giacomo Buscemi

**Affiliations:** Istituto di Genetica Molecolare “Luigi Luca Cavalli-Sforza”, CNR, Pavia, 27100 Italy

**Keywords:** R-loops, Senataxin, DNA damage, Micronuclei, cGAS

## Abstract

Senataxin is an evolutionarily conserved DNA/RNA helicase, whose dysfunctions are linked to neurodegeneration and cancer. A main activity of this protein is the removal of R-loops, which are nucleic acid structures capable to promote DNA damage and replication stress. Here we found that Senataxin deficiency causes the release of damaged DNA into extranuclear bodies, called micronuclei, triggering the massive recruitment of cGAS, the apical sensor of the innate immunity pathway, and the downstream stimulation of interferon genes. Such cGAS-positive micronuclei are characterized by defective membrane envelope and are particularly abundant in cycling cells lacking Senataxin, but not after exposure to a DNA breaking agent or in absence of the tumor suppressor BRCA1 protein, a partner of Senataxin in R-loop removal. Micronuclei with a discontinuous membrane are normally cleared by autophagy, a process that we show is impaired in Senataxin-deficient cells. The formation of Senataxin-dependent inflamed micronuclei is promoted by the persistence of nuclear R-loops stimulated by the DSIF transcription elongation complex and the engagement of EXO1 nuclease activity on nuclear DNA. Coherently, high levels of EXO1 result in poor prognosis in a subset of tumors lacking Senataxin expression. Hence, R-loop homeostasis impairment, together with autophagy failure and unscheduled EXO1 activity, elicits innate immune response through micronuclei formation in cells lacking Senataxin.

**Supplementary Information:**

The online version contains supplementary material available at 10.1007/s00018-024-05380-3.

## Introduction

Senataxin is an evolutionarily conserved DNA/RNA helicase with anti-R-loop activity [1, 2]. R-loops are three-stranded nucleic acid structures composed of a DNA:RNA hybrid and a displaced single-stranded DNA, which are physiologically produced during transcription by re-invasion of nascent RNA into the DNA template [3–5]. While R-loop formation is crucial to regulate gene expression, their unscheduled accumulation can interfere with DNA replication and repair, thus promoting DNA damage, replication stress and genome instability [3–5]. R-loops have been therefore described as one of the most important sources of DNA damage in tumorigenesis. Senataxin cooperates with the breast and ovarian cancer BRCA1 protein in the removal of R-loop-driven DNA damage at transcribed genes [6] and was found down-regulated in a range of cancer types, including ovarian cancer, suggesting a tumor suppressor activity [1]. Mutations in Senataxin gene (*SETX*) also cause two distinct juvenile-onset hereditary neurological diseases, Ataxia with Oculomotor Apraxia type 2 (AOA2) [7] and Amyotrophic lateral sclerosis type 4 (ALS4) [8], and in addition, defects in Senataxin function have been associated with the neuromuscular disorder Spinal Muscular Atrophy (SMA) [9]. Deregulation of R-loop homeostasis is not only observable in cancer but is also a sign of neurodegeneration, characterizing Senataxin-associated neurological diseases among the others. Thus, the study of R-loop-dependent dysfunctions in Senataxin-deficient cells is paradigmatic for our understanding of the negative impact of these structures in both cancer and neurodegeneration.

A still largely uncharacterized outcome of DNA damage and chromosomal instability in human cells is the formation of micronuclei (MN), which originate from lagging chromosomes or acentric fragments wrapped into a separate, and in some cases abnormal, nuclear envelope [10]. Initially associated with the disruption of the spindle assembly [11], MN formation was recently described to be triggered also by nuclear DNA damage, including that driven by unscheduled R-loop accumulation [12, 13]. Notably, chromosome fragments inside MN could derive from a defective repair of specific DNA lesions, among which double strand breaks (DSBs) are particularly harmful. MN are validated biomarkers of genome pathology associated with a wide range of developmental and degenerative diseases, including accelerated ageing, inflammation and neurodegeneration [14]. Recently, it was demonstrated that the cytosolic sensing pathway that initiates innate immune response to exogenous, and potentially pathogenic, DNA was also able to recognize self-DNA in a MN with a broken membrane [15]. Particularly, the central sensor of this pathway, cGAS, can bind to cytoplasmic DNA inside MN, as observed with drugs that target mitosis [15], upon radiotherapy [16], DNA damaging agents exposure [15, 17] and in mouse cells deficient for RNase H2, the enzyme mutated in autoimmune disorder Aicardi-Goutières syndrome (AGS) [18]. Furthermore, in line with the strong association between inflammation and cancer, cGAS activation by MN has been linked to cancer progression, promoting cellular invasion and metastasis [19].

Recent observations showed that high level of R-loops can trigger innate immune response in different contexts, including cellular model of AGS [20, 21], aged pancreas [22] and zebrafish hematopoietic stem and progenitor cells [23], although the mechanism involved remains mostly unexplored. Moreover in Senataxin- or BRCA1-deficient cells, RNA:DNA hybrids excided from nuclear R-loops by XPG/XPF nucleases, once released in the cytoplasm via a nuclear membrane export mechanism, were suggested to be directly sensed by the cGAS protein [24]. However, while emerging to be crucial for several human diseases, the pathological link between R-loops and inflammation, and in particular the role played by MN in the process, needs further investigations to be fully understood.

Here, we report that Senataxin deficiency induces the formation of MN in cancer cells, in the absence of any other stress condition. These Senataxin-dependent MN, differently from those deriving from genotoxic events or BRCA1 loss, display defects in membrane integrity and are prone to recruit the cGAS protein. This subtype of cGAS-positive MN is normally degraded by autophagy, a process which is impaired in Senataxin-deficient cells. The formation of these immunogenic MN and the downstream activation of interferon-stimulated genes, depend on persistent R-loops promoted by the RNA polymerase II processivity factor SPT4 and EXO1 nuclease activity, thus unravelling a novel R-loop-dependent pathway of cGAS activation.

## Materials and methods

### Cell culture

Human cell lines U2OS (osteosarcoma), MG-63 (osteosarcoma), MCF-7 (breast cancer), BJ-hTERT (normal fibroblast) were obtained from the American Type Culture Collection (ATCC) or from the European Collection of Authenticated Cell Cultures (ECACC) and periodically tested for mycoplasma contamination. U2OS, MCF-7 and BJ-hTERT were cultured in DMEM (Lonza) supplemented with 10% fetal bovine serum (FBS), 100 U/ml penicillin and 0.1 mg/ml streptomycin; MG-63 was maintained in MEM (Lonza) supplemented with 10% FBS, 100 U/ml penicillin and 0.1 mg/ml streptomycin. All cell lines were maintained at 37 °C and 5% CO2. For serum starvation MG-63 cells, after silencing with CTRL or SETX siRNA, were grown for 48 h in MEM medium without FBS.

### Cells transfections and treatments

Plasmids, herring sperm DNA (Sigma-Aldrich) and siRNAs transfections were carried out using Lipofectamine 2000 and RNAiMAX (Thermo Fisher Scientific), respectively, according to the manufacturers’ instructions. pEGFP-RNaseH1 plasmid was obtained from Addgene repository (plasmid # 108699; http://n2t.net/addgene:108699; RRID: Addgene_108699) [25]. Two rounds of silencing at 24 h distance were performed for SETX (respectively with 30 and 60 nM siRNA). Nucleases siRNAs were transfected 24 h before and re-transfected with siSETX. Cells were analyzed 48–96 h after the first round of silencing. siRNAs sequences are listed in Supplementary Table 1. Neocarzinostatin (Merck) treatment was performed at a concentration of 16 nM, chloroquine at 15 µM, RU.521 at 20 µM.

### Immunofluorescence

MN were identified by DAPI staining and assessed from random fields of view at microscope observation. Only interphase, non-apoptotic cells were analyzed (cells containing > 3 MN were excluded to further minimize the possibility of including apoptotic cells). MN were defined by previously published criteria [26]; essentially they should be separated from the primary nucleus, displaying a round or oval shape, their diameter should not exceed 1/3 than that of the primary nucleus with an intensity of DAPI staining similar or, occasionally, more intense. MN were enumerated during microscope observation in 100–1000 cells per experimental condition in minimum three independent experiments. Immunofluorescence stainings for cGAS, Rb and γ-H2AX were performed as previously described [27]. Essentially, cells grown on coverslips were fixed with 4% paraformaldehyde, permeabilized with 0.5% Triton X-100 in PBS, blocked in 3% BSA in PBS, stained with specific primary antibodies (see Supplementary Table 1) and Alexa Fluor 488, 555 or 647 conjugated secondary antibodies. MN identified first by DAPI staining were then scored for the presence of an intense cGAS staining. MN were considered Rb positive, based on visual assessment, if the fluorescence intensity present in the micronucleus was similar to the primary nucleus. For pRPA32 S4/S8 staining a pre-extraction step (0.5% Triton X-100 in PBS for 2’ on ice) was added before fixation. For S-phase analyses, EdU staining with Click-iT-EdU Alexa Fluor 488 (Thermofisher) was performed as previously described [28]. Mitotic figures were evaluated by DAPI staining [28]. DNA:RNA hybrids detection was performed essentially as previously described [29]. Briefly, cells grown on coverslips were fixed with 4% paraformaldehyde, permeabilized with 0.5% Triton X-100 in PBS, blocked in 3% BSA, 0.1% Tween20, 4x SSC, incubated with specific primary antibodies (S9.6 and DDX24, see Supplementary Table 1) and Alexa Fluor 488 or Alexa Fluor 555 conjugated secondary antibodies and DAPI. As a negative control, a slide was treated in situ with 50 U/ml of RNase H (New England Biolabs) for 2.5 h at 37 °C. Images were acquired using Zeiss AxioImager M2 microscope. Mean S9.6-associated fluorescence intensity was automatically evaluated inside nuclei (identified by DAPI staining), excluding nucleoli regions (DDX24 positive) using an ImageJ software pipeline. For IRF3 staining MG-63 cells were exposed for 6 h to the proteasome inhibitor MG132 (2.5 µM) to prevent the proteasome-dependent negative loop that shuts down the activation of this protein [30].

### Western blot

Western blot analyses were performed on total cell extracts using the NuPAGE system (Thermo Fisher Scientific) or the Mini PROTEAN TGX gels and the Trans-Blot Turbo Transfer System (Biorad). Antibodies used are listed in Supplementary Table 1.

### RT-qPCR

Total RNA was extracted using the RNeasy Mini Kit (QIAGEN), according to manufacturer’s instructions and quantified using NanoPhotometer P330 (Implen). 1 µg of total RNA was retro-transcribed using the SuperScript IV First-Strand Synthesis System (Thermo Fisher Scientific). qPCR was performed in triplicate on 20 ng of cDNA using QuantiFast SYBR Green PCR Kit (Qiagen) and the LightCycler 480 System (Roche). Reactions were performed in 20 µl of final volume in triplicates. Samples were normalized using GAPDH as reference gene. Primer sequences are listed in Supplementary Table 1.

### Crystal violet assay

MG-63 cells were transfected with specific siRNAs and seeded in triplicates in 6 wells plates. 6–8 days later, cells were stained with crystal violet solution. After washing, the plates were dried for 24 h and successively, the staining was eluted with 1% SDS until the crystal violet was completely dissolved. The photometric absorbance of the solution was measured at 570 nm using a microplate reader (Tecan, Crailsheim, Germany).

### Clinical datasets analysis

The website cBioPortal (http://www.cbioportal.org) was used for meta-analysis of the association between SETX or EXO1 expression levels and prognosis. Patients from different TCGA cohorts (Ovarian serous cystadenocarcinoma, Head and Neck Squamous cell Carcinoma, Brain Lower Grade Glioma) were stratified based on SETX and EXO1 z-score and the first and last deciles were identified as high and low expression groups. Only patients displaying all the considered parameters (SETX and EXO1 expression, survival status, overall survival) were included. Kaplan-Meier plots for overall survival data were performed in Prism 5 (GraphPad) and significance was determined using log-rank test.

## Results

### cGAS positive micronuclei accumulate in Senataxin-deficient cycling cells

**Fig. 1:**
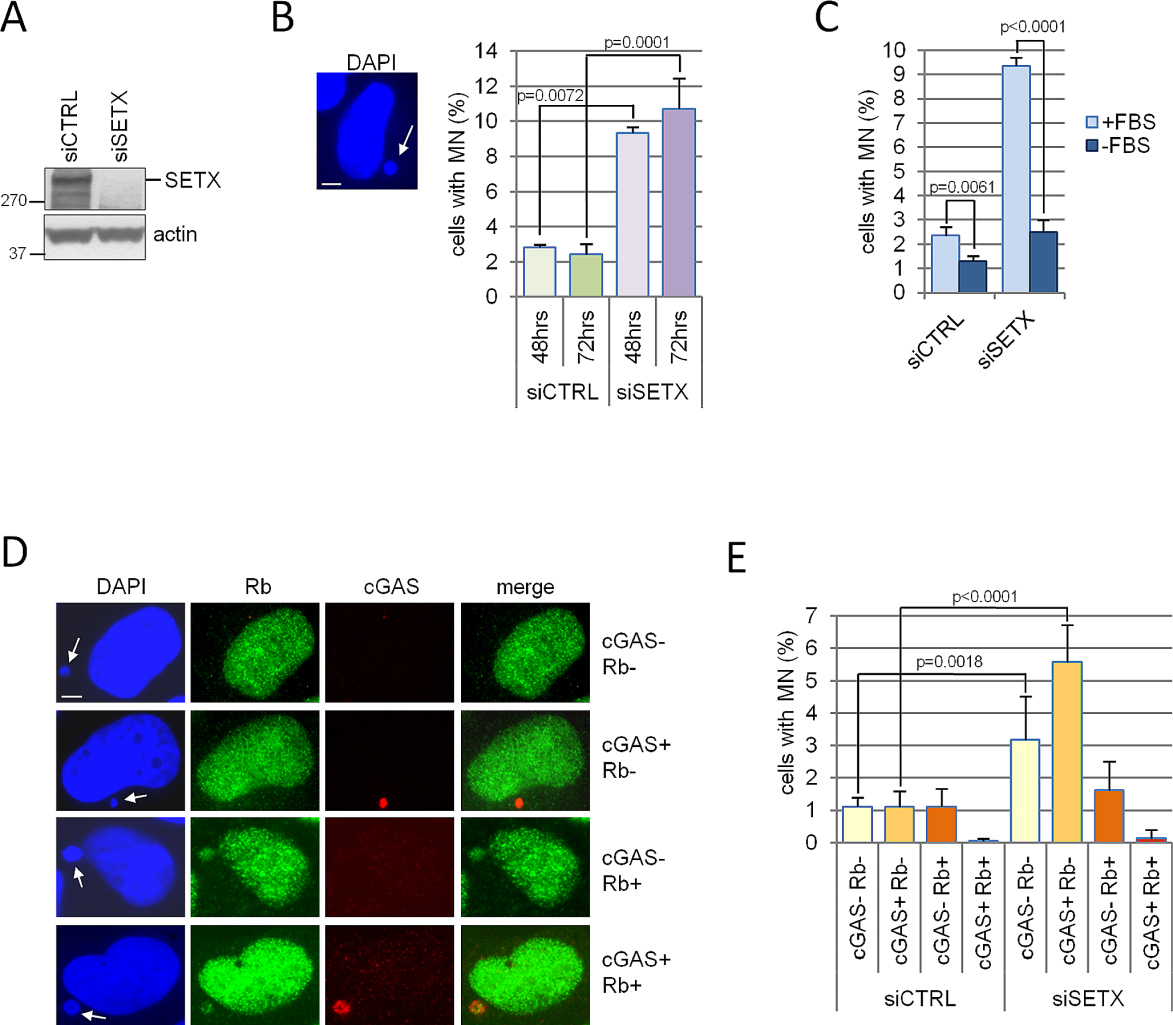
cGAS positive MN accumulate in Senataxin-deficient cycling cells. **(A)** U2OS cells were transfected with control (siCTRL) or SETX siRNA. Senataxin protein levels were assessed by western blot at 72 h after transfection. Actin was used as loading control. **(B)** U2OS cells silenced for CTRL or SETX were fixed at the indicated time points and stained with DAPI to reveal MN (white arrow). In the image brightness/contrast levels were adjusted to point out the micronucleus without altering the data. White bar = 5 μm. Cells with a micronucleus were enumerated scoring at least 500 nuclei. The graph shows the mean and standard deviation (s.d.) of three biologically independent experiments. The exact *p* value was obtained using a two-tailed unpaired Student’s t test. **(C)** MG-63 cells after silencing with CTRL or SETX siRNA, were grown for 48 h in medium supplemented (+ FBS) or not with serum (-FBS). Cells were successively fixed and MN-positive cells enumerated by DAPI staining. The graph shows the mean and s.d. of three biologically independent experiments. The exact *p* value was obtained using a two-tailed unpaired Student’s t test. **(D)** Representative images showing localization of cGAS (red) and Rb (green) inside MN (DAPI, blue, white arrow), detected by immunofluorescence using specific primary antibodies. White bar = 5 μm. In the images brightness/contrast levels were adjusted to point out the micronucleus without altering the data. **(E)** Graph shows data obtained by counting immunostained U2OS cells silenced or not (siCTRL) for SETX. Bars represent the percentage (mean and s.d.) of cells in the population characterized by a micronucleus negative for both cGAS and Rb (cGAS- Rb-), positive for cGAS alone (cGAS+ Rb-), positive for Rb alone (cGAS- Rb+), positive for both (cGAS+ Rb+). Frequencies were obtained scoring 500 nuclei and performing at least five biologically independent experiments. The exact *p* values obtained using a two-tailed unpaired Student’s t test are shown only for statistically significant comparisons (*p* < 0.05)

To investigate the genome instability phenotypes associated with SETX-deficiency, we performed an efficient silencing of SETX in different human cell lines (U2OS, MG-63, MCF-7 and BJ-hTERT; Fig. 1A and Supplementary Fig S1A). Staining the DNA, we observed that, compared to control, siSETX cells, although not exposed to any genotoxic treatment, are characterized by 2 to 4 folds MN accumulation in the cytoplasm (Fig. 1B and Supplementary Fig S1B). Particularly, in cancer cell lines, MN are detectable in the cytoplasm of up to 11% of SETX depleted cells and of up to 4% of siCTRL transfected cells (Fig. 1B and Supplementary Fig S1B).

The accumulation of MN in the absence of Senataxin depends on cell cycle progression as it was not observed upon serum starvation (Fig. 1C and Supplementary Fig S1C) and occurs even if siSETX cells are slightly slow growth (Supplementary Fig S1D). Similar results were obtained using an alternative siRNA against SETX (Supplementary Fig S1E and F).

MN may be characterized by a discontinuous membrane that allows the recruitment of the double strand DNA binding protein cGAS, the apical sensor of the innate immune response pathway [15], and MN chromatin content was also found to influence DNA recognition by the cGAS protein [31]. Therefore, we investigated whether the MN accumulating in Senataxin deficient cells are capable to recruit cGAS. For this purpose, we immunostained control and SETX silenced cells with specific antibodies against cGAS and Rb, the latter used as a marker of membrane integrity as it is retained in MN with an unbroken membrane [10]. As expected, we found that Rb positive MN are essentially cGAS negative in both control and siSETX cells, (Fig. 1D and E). On the other side, the fraction of Rb negative and cGAS positive MN is significantly higher in siSETX cells compared to siCTRL (Fig. 1E and Supplementary Fig S1G). In fact, in Senataxin-deficient samples 5.6% of the cells has a cGAS-positive micronucleus compared to 1% of CTRL cells (Fig. 1E). Similar distributions were obtained in all the tested cell lines (Supplementary Fig S1H) and employing an alternative siRNA for SETX (Supplementary Fig S1I). Taken together, these data demonstrate that Senataxin depletion promotes the accumulation of MN characterized by a defective membrane and prone to be recognized by the cGAS protein.

MN formation is a cell cycle-dependent event (Fig. 1C, [10]), as well as micronuclear membrane rupture, at least in some experimental conditions [32]. Therefore, to rule out that cGAS-positive MN accumulation could be a consequence of an altered cell cycle progression due to Senataxin depletion, we stained (Supplementary Fig S1J) our cells at the same time for cGAS, cyclin B1 (to mark G2 cells) and DNA synthesis (EdU, to detect S phase cells). In siCTRL, the MN positive cells distribution shows a modest accumulation in S phase compared to the general population (Supplementary Fig S1K), while cGAS positive MN were more frequent in S phase cells (Supplementary Fig S1K). A similar behaviour was detected in siSETX cells, with MN positive population overlapping the general distribution of cells throughout the cell cycle, and an increased frequency of cGAS positive MN in S phase (Supplementary Fig S1K), while siSETX have actually fewer cells in replication than siCTRL (Supplementary Fig S1D). Therefore, the increased frequency of cGAS positive MN in siSETX cells is not due to a peculiar cell cycle distribution, although they accumulate preferentially in S phase.

### The accumulation of cGAS positive micronuclei depends on co-transcriptional R-loop persistence and impaired autophagy

**Fig. 2:**
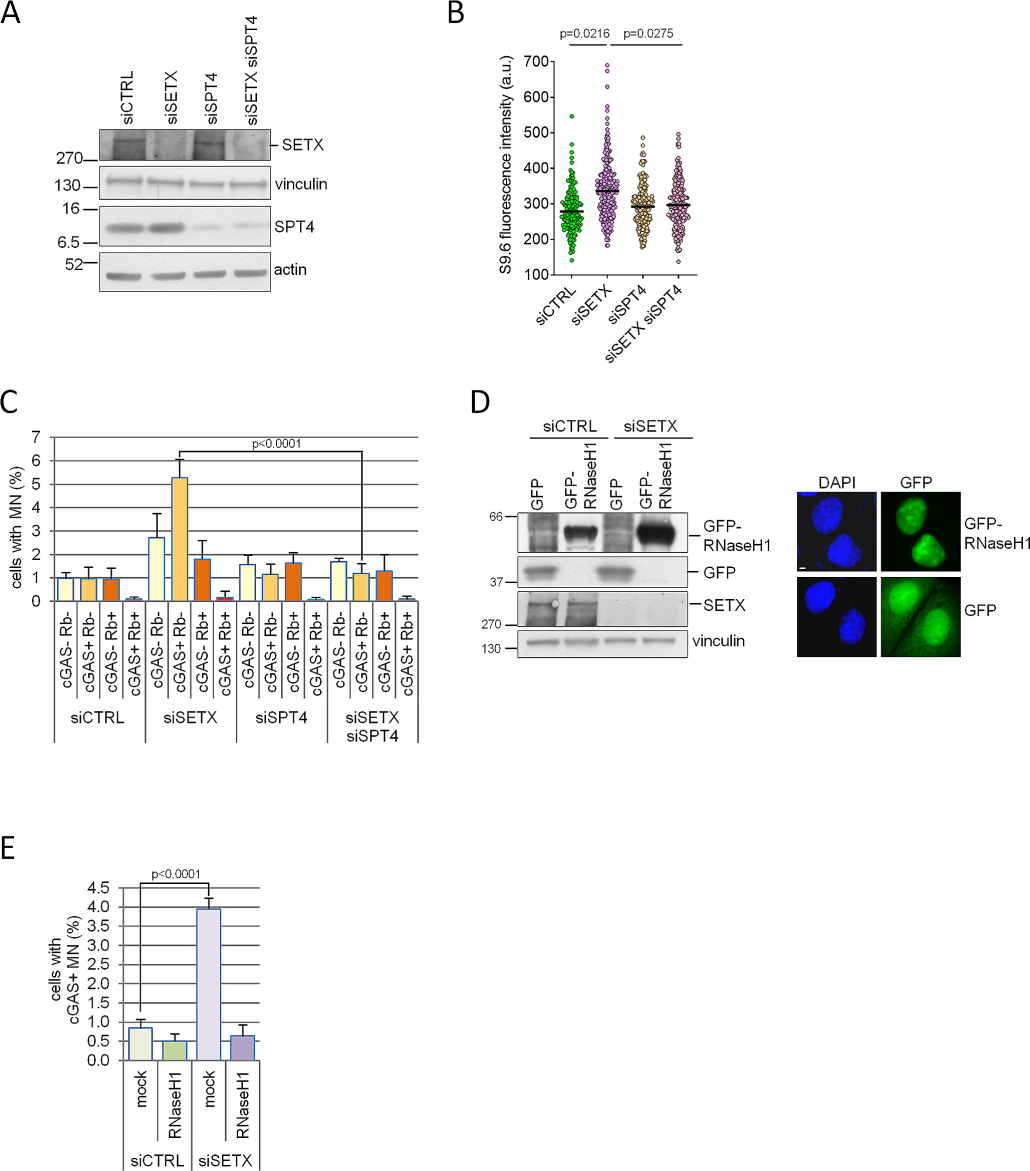
Transcription elongation and persistent R-loops promote cGAS accumulation in MN of Senataxin-deficient cells. **(A)** Western blot analysis of U2OS cells transfected with control (siCTRL) or SETX, SPT4 and SETX + SPT4 siRNAs. Senataxin and SPT4 protein levels were assessed 72 h after transfection. Vinculin and actin were used as loading control. **(B)** RNA:DNA hybrids were assessed by immunostaining with S9.6 antibody in cells transfected as in (A). Pictures were acquired for each sample and the intensity of the signal in the nucleus, excluding nucleoli, was quantified for 30 cells. The signal intensity was expressed in an arbitrary unit (a. u.) for each cell deriving from three biologically independent experiments. The median is included in the graph and the *p* values were obtained by one way ANOVA plus Bonferroni post hoc test. **(C)** Cells as in (A) were immunostained with cGAS and Rb antibodies. MN were scored as positive for the presence of one or both proteins. The graph shows the mean and s.d. of four biologically independent experiments. The exact *p* values were obtained using a two-tailed unpaired Student’s t test and shown only for statistically significant comparisons between siSETX and siSETX siSPT4 data. **(D)** U2OS cells were transfected with control (siCTRL) or SETX siRNAs and, successively, with plasmids expressing GFP or GFP-RNase H1. GFP-RNase H1 protein expression was tested 48 h after transfection by western blot using antibodies against GFP and vinculin, as loading control (left). In parallel cells were visualized to verify the presence of GFP signal (right, white bar = 5 μm). **(E)** Cells (GFP = mock and GFP-RNase H1 = RNase H1) as in (D) were tested by immunostaining for the presence of cGAS positive (cGAS+) MN. Bars represent means with s.d. obtained scoring 500 nuclei for each of three biologically independent experiments. The exact *p* values were obtained using the one way ANOVA plus Bonferroni post hoc test

In Senataxin deficient cells MN formation can be a consequence of R-loop persistence due to transcription stress, resulting in DNA breakage, an event known to promote micronucleation. Recently, the attenuation of RNA polymerase II elongation activity, caused by the knock-down of SPTs proteins, was found to suppress R-loop accumulation and R-loop-driven DNA damage due to SETX deficiency in yeast [33]. This evidence is particularly intriguing since the slow-down of transcription was, in other context, considered an event increasing R-loop frequency [34, 35]. Therefore we decided to evaluate whether depletion of SPT4, an accessorial component of the elongation complex DSIF (DRB Sensitivity-Inducing Factor) [36], was able to suppress R-loop accumulation due to Senataxin depletion, as well as the accumulation of cGAS positive MN. A reduction of more than 80% of SPT4 protein was obtained by silencing (Fig. 2A) without affecting cell growth in U2OS (Supplementary Fig S2A). Coherently with observations made in yeast, SPT4 knock-down (KD) was able to suppress almost completely SETX-dependent accumulation of RNase H-sensitive RNA:DNA hybrids as demonstrated by immunostaining with S9.6 antibody (Fig. 2B and Supplementary Fig S2B). Moreover, SPT4 silencing strongly suppresses the increase of MN (Supplementary Fig S2C), including the fraction of cGAS positive MN (Fig. 2C) associated with SETX depletion. A similar SPT4-mediated suppression of MN was observed in other cell lines (Supplementary Fig S2D-F). Thus, in Senataxin-deficient cells, the formation of both R-loops and cGAS-positive MN is due to transcription stress generated during RNA polymerase II elongation.

To demonstrate the existence of a direct link between the accumulation of MN and R-loops in SETX depleted cells, we overexpressed a GFP tagged form of RNase H1 (Fig. 2D), which is known to efficiently remove R-loops [37], in control and SETX silenced cells. We observed that the expression of RNase H1 in siSETX cells (Fig. 2D) was sufficient to reduce MN (Supplementary Fig S2G), including the fraction of cGAS-positive MN (Fig. 2E). Taken together, our findings demonstrate that R-loops, resulting from transcription stress, trigger MN accumulation in the absence of Senataxin.

**Fig. 3:**
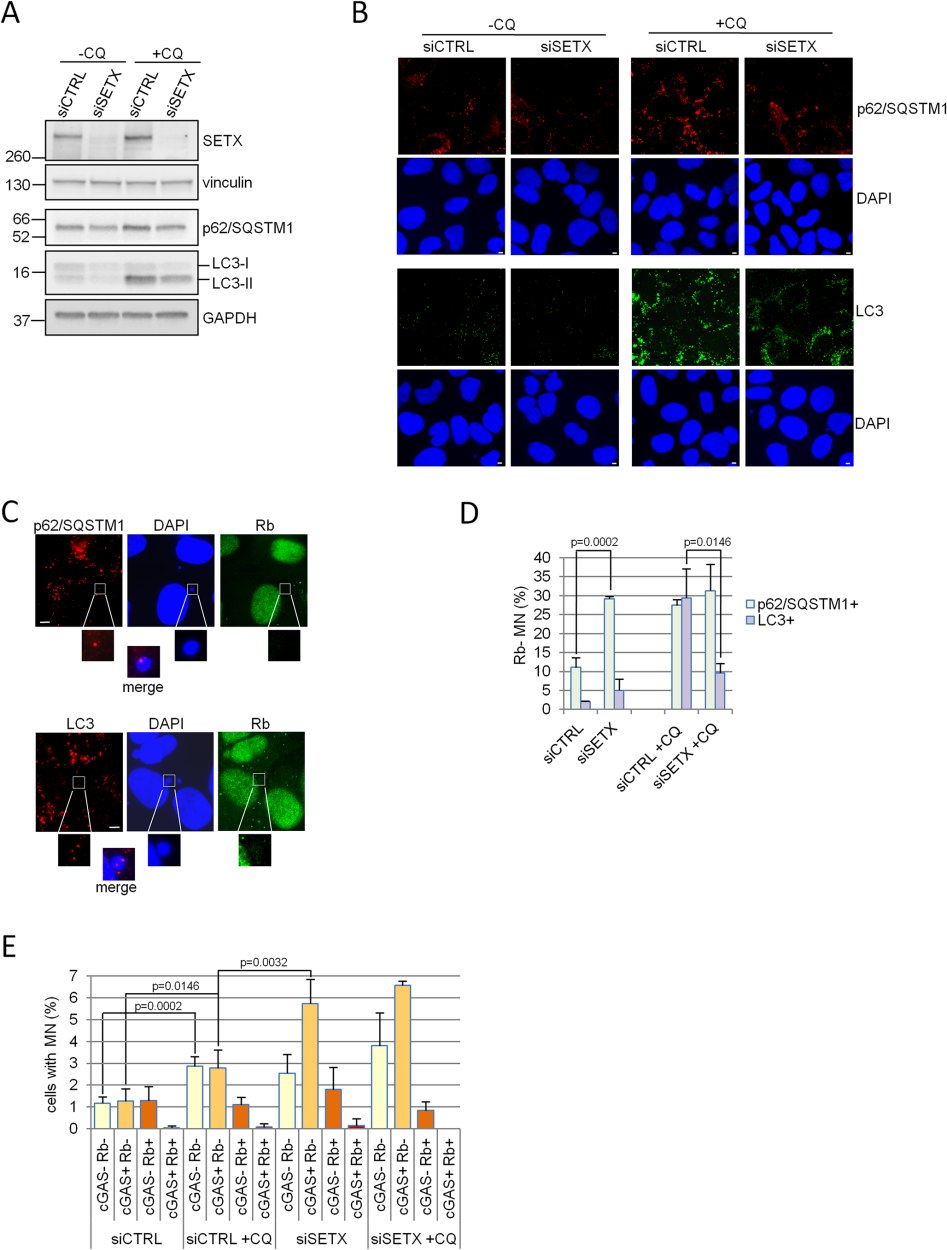
Autophagy impairment contributes to membrane defective and cGAS positive MN accumulation in Senataxin-deficient cells. **(A)** Cells silenced with siCTRL and siSETX were treated for 2 h with 40 µM Chloroquine (CQ) and levels of the indicated proteins were tested by western blot. Vinculin and GAPDH were used as loading control. **(B)** Cells silenced with siCTRL and siSETX were treated for 24 h with 15 µM CQ, fixed and assayed by immunofluorescence for p62/SQSTM1 and LC3 protein expression and localization. DAPI counterstains nuclei. **(C)** Representative images of p62/SQSTM1 and LC3 puncta colocalizing with Rb negative MN in siCTRL cells treated with CQ (as in B). White bar = 5 μm (B and C panels). In B and C panels image brightness/contrast levels were adjusted to point out p62/SQSTM1 and LC3 protein foci and the micronucleus without altering the data. **(D)** Bars represent the fraction of Rb negative MN marked by p62/SQSTM1 (p62/SQSTM1+) or LC3 (LC3+) puncta in siCTRL or siSETX cells treated or not with CQ. At least 30 MN were evaluated for each condition in three biologically independent experiments. Bars represent means with s.d.; the exact *p* values were obtained using a two-tailed unpaired Student’s t test and shown only for statistically significant comparisons between siSETX and siSETX or siCTRL + CQ and siSETX + CQ samples. **(E)** Cells silenced with siCTRL and siSETX were treated for 30 h with 15 µM CQ, fixed, stained with DAPI and immunostained with cGAS and Rb. MN were scored as positive for the presence of one or both proteins. Data were obtained scoring at least 500 nuclei and from five biologically independent experiments. Bars represent means with s.d.; the exact *p* values were obtained using a two-tailed unpaired Student’s t test; no statistically significant differences were obtained comparing the corresponding categories in siSETX and siSETX + CQ samples

Since MN are subjected to degradation by autophagy [38] and Senataxin deficient cells showed reduced autophagic activity [39], we reasoned that an impairment of this process may contribute to MN accumulation. To explore this possibility, we initially tested the levels of p62/SQSTM1, an autophagy receptor, and of LC3-I/II, an adaptor protein that in the LC3-II form promotes targets recruitment to the autophagosome. Both these proteins are degraded during autophagy and have been previously involved in the autophagic clearance of MN [40]. We found that in siSETX cells p62/SQSTM1 and LC3 are both reduced compared to siCTRL (Fig. 3A and Supplementary S3A). Moreover, the exposure to chloroquine (CQ), that inhibits the final stages of autophagy, and therefore p62/SQSTM1 and LC3 degradation, induces the accumulation of p62/SQSTM1 and LC3-II in siCTRL, but only partially in siSETX cells (Fig. 3A and Supplementary S3A), demonstrating that the autophagic pathway is altered in the absence of SETX. These data were further confirmed by immunofluorescence staining that revealed a punctuate pattern for both these proteins, indicating their translocation to the autophagosomal membranes: a reduced signal in siSETX cells, compared to siCTRL, was indeed observed both before and after CQ exposure (Fig. 3B and Supplementary S3B). To verify whether these autophagic markers are targeted to membrane-defective MN, we analyzed the co-localization of p62/SQSTM1 and LC3 puncta with Rb negative MN (Fig. 3C). We found that 11.1% of the Rb negative MN were positive for p62/SQSTM1 puncta and 2% were positive for LC3 puncta (Fig. 3D). This colocalization does not occur by chance, since we were unable to find any Rb positive MN marked by the presence of p62/SQSTM1 or LC3 puncta (Supplementary Fig S3C). In siSETX cells, even though the total signal of p62/SQSTM1 is slightly reduced, we found a significant increase of p62/SQSTM1 positive/Rb negative MN (up to 30%) and only a slight, not statistically significant, increase of colocalization with LC3 (Fig. 3D). These data suggest that, in control cells, only a small percentage of membrane-defective MN characterized by a defective membrane colocalized with markers of autophagy, an observation coherent with previously published data [40]. Conversely, in siSETX cells the same class of MN is strongly positive for p62/SQSTM1 but not for LC3 (Fig. 3D). These results therefore suggest that the recognition of MN is efficient in cells deprived of SETX, but not their clearance. This conclusion was further confirmed blocking autophagic degradation turnover by CQ treatment. Indeed, in this condition about 30% of the Rb negative MN became positive for p62/SQSTM1 or LC3 in siCTRL cells, while no significant increase of LC3 positive MN can be observed in siSETX cells (Fig. 3D). Altogether our data suggest that a specific step in the autophagic clearance of membrane-defective MN is impaired in SETX depleted cells.

Since p62/SQSTM1 was previously found to bind micronuclear cGAS [38], we hypothesized that autophagy deregulation could be at least partially responsible for the increase of cGAS positive MN in these cells. To explore this possibility, we enumerated MN in control and siSETX cells exposed to CQ and we noticed that while in control cells the frequency of MN increases more than two-fold after CQ, the same effect cannot be observed in siSETX cells (Supplementary Fig S3D). However, MN frequency in control cells treated with CQ is still half than that observed in siSETX cells (Supplementary Fig S3D). These results reinforce the notion that the autophagic-mediated clearance of MN is impaired in siSETX cells, since CQ treatment does not affect the accumulation of these structures in Senataxin-deficient cells.

Notably, we found that control cells treated with CQ mainly accumulate Rb negative MN (Fig. 3E), demonstrating that autophagy acts preferentially on ruptured MN. Since half of the Rb negative MN were cGAS positive (Fig. 3E), these findings suggest that defects in autophagy could contribute preferentially to the accumulation of cGAS positive MN. In accordance with an impairment of the autophagic process, in siSETX cells CQ exposure does not significantly affect cGAS positive MN frequency (Fig. 3E).

Overall, these data highlight that two mechanisms contribute to cGAS positive MN accumulation in unstressed Senataxin deficient cells: an increased production deriving from genome instability associated with persistence of co-transcriptional R-loops and a reduced degradation caused by autophagy defects.

### Senataxin-dependent micronuclei are specifically prone to cGAS recruitment, contain damaged DNA and their formation is promoted by EXO1 nuclease

**Fig. 4:**
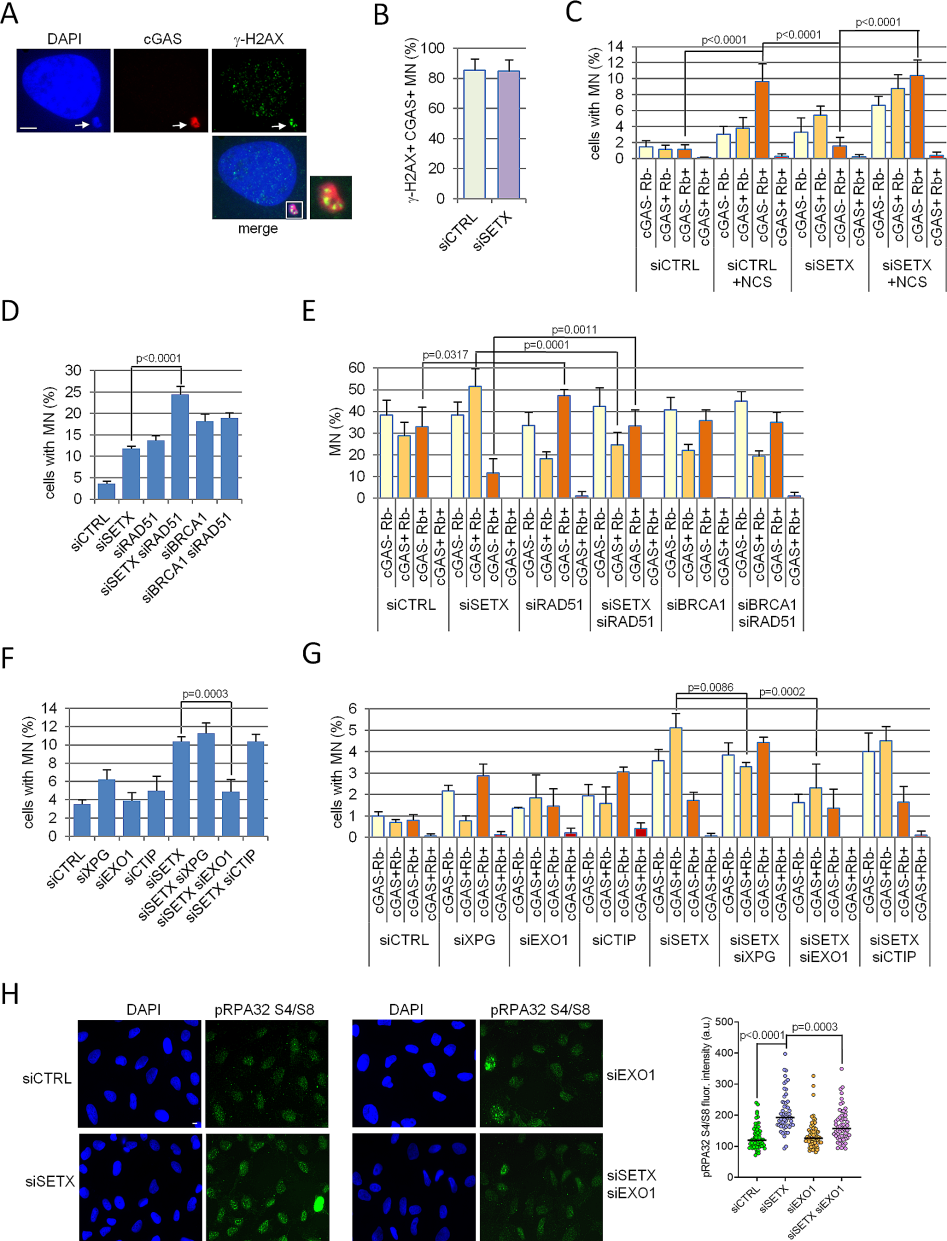
Senataxin-dependent MN are specifically prone to cGAS recruitment, contain damaged DNA and their formation is promoted by EXO1 nuclease. **(A)** Representative immunofluorescence images showing co-localization of cGAS (red) and γ-H2AX (green) inside MN (blue, white arrow) of U2OS cells. DNA is stained with DAPI. In the image brightness/contrast levels were adjusted to point out the micronucleus without altering the data. **(B)** Graph represents the fraction of cGAS positive (cGAS+) MN showing γ-H2AX foci (γ-H2AX+). The values in the graph are mean ± s.d. of three biologically independent experiments. The difference between the two samples is not significant according to a two-tailed unpaired Student’s t test (*p* = 0.2178). **(C)** U2OS cells were transfected with siCTRL or siSETX and exposed to 16 nM of neocarzinostatin (NCS) for 24 h, fixed, stained and evaluated for cGAS and Rb positivity. The values in the graph are mean ± s.d. of four biologically independent experiments. The exact *p* values were obtained comparing exclusively the cGAS- Rb+ category of each condition and using the one way ANOVA plus Bonferroni post hoc test. **(D)** U2OS cells silenced for the indicated combination of genes were evaluated for MN production by DAPI staining. The values in the graph are mean ± s.d. of three biologically independent experiments. The exact *p* values were obtained using two-tailed unpaired Student’s t test; no statistically significant differences were obtained comparing siBRCA1 with siBRCA1/siRAD51 samples. **(E)** Cells transfected with the indicated siRNAs were analyzed 72 h after transfection by immunostaining for cGAS and Rb positivity. The values in the graph are mean ± s.d. of three biologically independent experiments. The exact *p* values were obtained using two-tailed unpaired Student’s t test; no statistically significant differences were obtained comparing siSETX/siRAD51 with siBRCA1 and siBRCA1 with siBRCA1/siRAD51. **(F)** U2OS cells silenced for the indicated genes were evaluated for MN production by DAPI staining. The values in the graph are mean ± s.d. of three biologically independent experiments. The exact *p* values were obtained using the two-tailed unpaired Student’s t test; no statistically significant differences were obtained comparing siSETX with siSETX/siXPG or siSETX/siCTIP. **(G)** siCTRL and siSETX cells pre-silenced with siRNAs against specific nucleases were evaluated for cGAS and Rb positivity 72 h after transfection. The values in the graph are mean ± s.d. of three biologically independent experiments and the *p* values were obtained by one way ANOVA plus Bonferroni post hoc test conducted exclusively on cGAS+ Rb- category of siSETX against double silencing samples. Only statistically significant differences are shown in the graph, the comparison between siSETX and siSETX siCTIP data result in a non significant difference (*p* = 0.7019). **(H)** Cells silenced with the indicated siRNAs were immunostained with an antibody against RPA32 phosphorylated at Serine 4 and 8 (pRPA32 S4/S8) and counterstained with DAPI to reveal nuclei. In the images brightness/contrast levels were adjusted to show pRPA32 S4/S8 signal without altering the data. White bar = 5 μm. The signal intensity in the nucleus was expressed in an arbitrary unit (a. u.) for each cell deriving from three biologically independent experiments. The median of the data is included in the graph and the *p* values were obtained by one way ANOVA plus Bonferroni post hoc test

MN contain whole chromosome or chromosome fragments that could be derived from unrepaired DNA breaks. The unscheduled persistence of R-loops during transcription was previously described as capable to induce DNA breaks [3] and mis-repairing events finally resulting in the accumulation of damaged DNA into MN. Coherently, in our experimental condition, the primary nucleus of siSETX cells accumulated more foci of the DNA damage marker γ-H2AX [41] than siCTRL (Supplementary Fig S4A left panel). On the other hand, MN (Supplementary Fig S4A right panel) and cGAS positive MN (Fig. 4A and B) showed similar frequency of γ-H2AX positivity in both siCTRL and siSETX cells, a percentage that reaches more than 80% in the case of cGAS positive MN (Fig. 4B). Thus, MN are likely produced mainly by DNA break events in both siCTRL and siSETX cells and cGAS positive MN are almost totally characterized by damaged DNA.

To evaluate if any MN produced by a DNA break is prone to cGAS recruitment, we treated U2OS cells with neocarzinostatin (NCS), an agent known to acutely induce DSBs [42]. We found that incubation with 16 nM of NCS promoted MN release in 18% of the cells (Supplementary Fig S4B) but with a distribution of cGAS/Rb staining different from that observed in siSETX cells. Indeed, NCS induces mainly (> 50%) Rb positive and cGAS negative MN, thus with an intact membrane (Fig. 4C and Supplementary Fig S4C). Even when SETX-depleted cells were exposed to NCS (Fig. 4C and Supplementary Fig S4C), we noticed an increase in the formation of MN, but they were not prone to recruit cGAS. This evidence suggests that the source of DNA damage leading to MN production influences the main traits of MN itself. To further confirm this hypothesis, we silenced RAD51 (Supplementary Fig S4D), a protein essential for DSB repair by homologous recombination [43] and we analyzed MN formation. As for NCS treatment, we found that MN accumulate in RAD51 depleted cells (Fig. 4D) but they are mainly cGAS negative and characterized by an intact membrane (Fig. 4E). In addition, combining SETX and RAD51 silencing, we observed significantly higher levels of MN (Fig. 4D) and an intermediate distribution of cGAS/Rb categories compared to that found upon the single gene silencing (Fig. 4E). Interestingly, the distribution of MN classes obtained in response to combined SETX and RAD51 silencing is comparable to that produced by the knock-down of BRCA1 (Supplementary Fig S4D and Fig. 4E), a protein with a role in DSBs repair by homologous recombination, replication stress and R-loop homeostasis [44]. Therefore these findings suggest that both SETX and BRCA1 depletions promote the formation of MN, but these structures differ for cGAS positivity likely because the majority of MN in BRCA1 deficient cells derive from DSB repair defects, while those in SETX silenced cells are due to defective R-loops processing. Accordingly, concomitant silencing of BRCA1 and RAD51, both impairing DNA repair, does not increase MN production (Fig. 4D) nor impacts on MN categories distribution (Fig. 4E). On the whole, these data demonstrate that, albeit the exposure to a DNA damaging agent and the depletion of SETX or BRCA1 can similarly induce DNA breaks, MN are generated through distinct mechanisms and have different features in those different contexts.

Since MN formation in the absence of Senataxin depends on R-loop accumulation and occurs in cycling cells, one hypothesis is that MN could result from the nuclease-mediated processing of replication forks colliding with R-loops. We therefore tested specific DNA nucleases that have been implicated in R-loop and/or fork processing. Depletion of XPG, a structure-specific endonuclease (Supplementary Fig S4E) that has been involved in R-loop processing and cytoplasmic RNA:DNA hybrids formation [24, 29], has no impact on MN formation (Fig. 4F) and only partially decreases cGAS positive MN accumulation in Senataxin-deficient cells (Fig. 4G). Similarly, the knock-down of CtIP (Supplementary Fig S4E), a factor implicated in DSB resection together with MRE11 nuclease [45] and also acting in R-loop incision [46], has no effect on Senataxin-dependent MN accumulation (Fig. 4F and G). Conversely, the silencing of EXO1 (Supplementary Fig S4E), a major nuclease processing DSBs and stalled replication forks [47, 48], significantly reduces Senataxin-dependent MN (Fig. 4F and G) without affecting cell cycle progression (Supplementary Fig S4F). EXO1 silencing has the same effect also in MCF-7 and MG-63 cells (Supplementary Fig S4G). Since the unscheduled resection activity of EXO1 in the nucleus could be responsible for the DNA damage leading to MN accumulation in SETX KD cells, we analized the levels of RPA32 phosphorylation at Ser4/Ser8 as a readout of such activity [49]. We indeed observed by immunofluorescence in SETX-depleted cells an increase in RPA32 phosphorylation at Ser4/Ser8 that is partially rescued by EXO1 co-depletion (Fig. 4H and Supplementary Fig S4H). These data indicate that the DNA contained in Senataxin-dependent MN is mainly due to a long-range resection associated with EXO1 activity, which could act on DNA extremities resulting from the processing of stalled replication forks and/or that of broken R-loops.

### cGAS positive micronuclei engage an EXO1- and SPT4-dependent activation of interferon genes in Senataxin-deficient cells

**Fig. 5:**
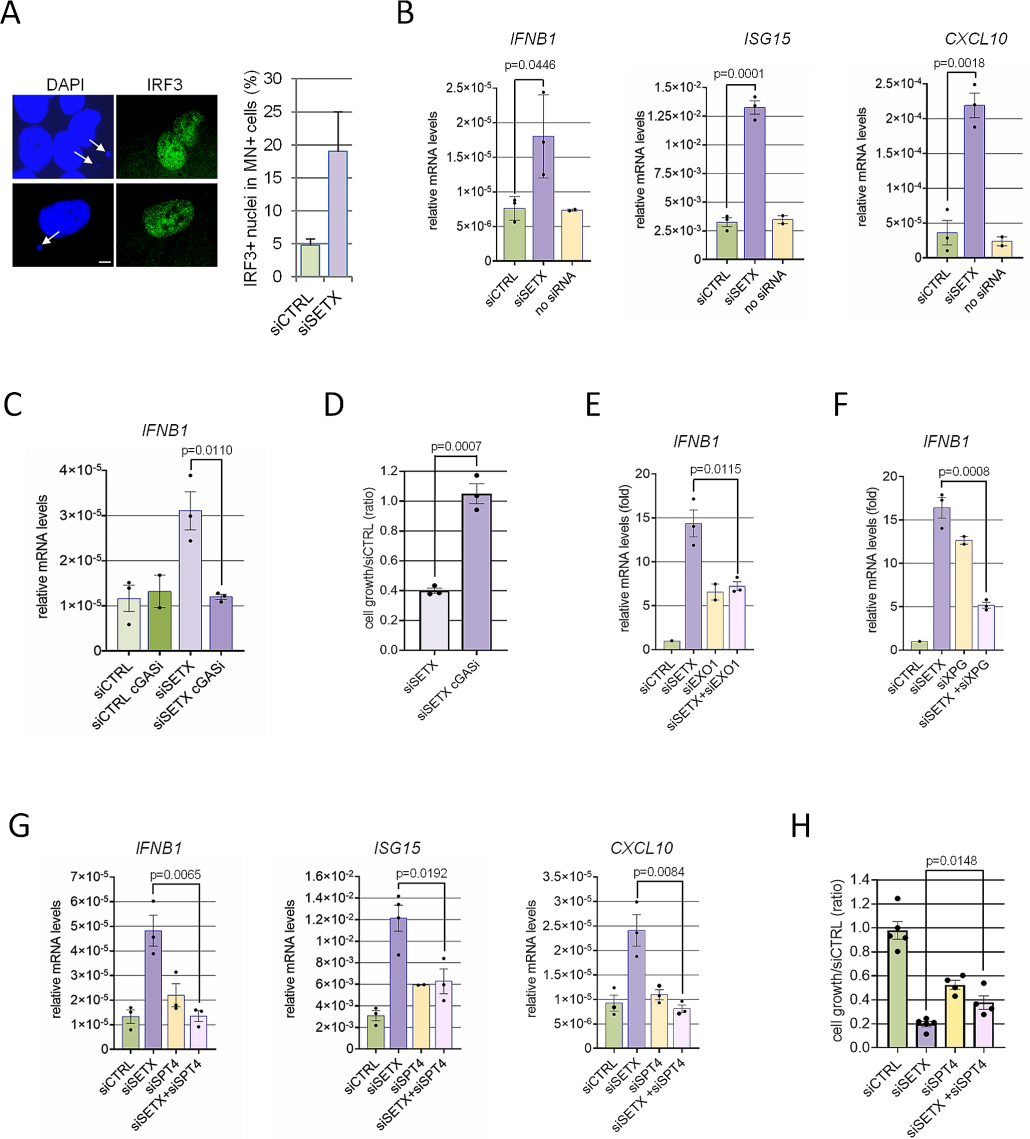
EXO1 and SPT4 promote the activation of interferon genes in Senataxin-deficient cells. **(A)** Representative immunofluorescence images showing IRF3 staining and localization in MG-63 cells with MN. DAPI was used to counterstain nuclei and MN (white arrows). White bar = 5 μm The fraction of siCTRL and siSETX cells positive for MN presence and characterized by IRF3 nuclear localization is depicted in the graph (mean ± s.d. of two biologically independent experiments). **(B)** RT-qPCR analysis of IFNB1, ISG15 and CXCL10 mRNAs expression levels (relative to GAPDH) in MG-63 cells transfected with siCTRL, siSETX or with no siRNA. mRNA was extracted from cells 48 h after a second round of silencing. The values in the graph are mean ± s.e.m. of three biologically independent experiments. The indicated exact *p* values were obtained using a two-tailed unpaired Student’s t test. **(C)** RT-qPCR analysis of IFNB1 mRNA levels in MG-63 cells transfected with siCTRL or siSETX and exposed for 48 h to 20 µM RU.521 (cGASi). The values in the graph are mean ± s.e.m. of three biologically independent experiments. The indicated exact *p* value was obtained using the two-tailed unpaired Student’s t test. **(D)** MG-63 cells silenced for siSETX were exposed to 20 µM RU.521 for 48 h, seeded as single cells and left to grow for 8 days. Cells were stained with crystal violet and the absorbance measured following dissolution of the dye. Data in the graph are siSETX/siCTRL ratio. Three independent experiments were performed, each seeding three different amounts of cells. In the graph means and s.e.m. are shown. The indicated exact *p* value was obtained using the two-tailed unpaired Student’s t test. (**E**) RT-qPCR analysis of IFNB1 mRNA levels in MG-63 cells pre-silenced with EXO1 siRNA and successively silenced for CTRL or SETX. The values in the graph are mean ± s.e.m. of three biologically independent experiments and the exact *p* value was obtained by two-tailed unpaired Student’s t test. **(F)** Same as (E) for siXPG pre-selencing. **(G)** RT-qPCR analysis of IFNB1, ISG15 and CXCL10 mRNA levels in MG-63 cells transfected with siCTRL, siSETX, siSPT4 or siSETX + siSPT4. mRNA was extracted from cells 48 h after silencing. The values in the graph are mean ± s.e.m. of three biologically independent experiments. The indicated exact *p* value was obtained using a two-tailed unpaired Student’s t test. **(H)** MG-63 cells silenced as in (G) were seeded as single cells and left to grow for 6 days. Cells were then stained with crystal violet and, after dissolution, the amount of dye was quantified by absorbance. Data in the graph were fold of siCTRL values. Three independent experiments were performed, each seeding three different amounts of cells. In the graph means and s.e.m. are shown and the exact *p* value was obtained using a two-tailed unpaired Student’s t test

Since cGAS accumulation inside MN was previously shown to induce the local activation of this protein [50, 51], we hypothesized that in siSETX cells a cGAS-dependent innate immune response was turned on. To address this point, we evaluated the subcellular localization of the interferon regulatory factor IRF3 whose phosphorylation and consequent translocation in the nucleus lead to interferon genes activation [30]. We found that IRF3 accumulates in the nuclei of those cells forming MN, which are more abundant in SETX-depleted cells (Fig. 5A). To further demonstrate that the accumulation of cGAS positive MN and the nuclear translocation of IRF3 in Senataxin depleted cells were indeed able to induce a complete interferon I response, we evaluated in our cells the transcription of IFNB1 and of two interferon-stimulated genes (ISG15 and CXCL10). We performed the experiments in MCF-7 and MG-63 cells since, differently from U2OS, they were previously described as capable to induce a full cGAS-interferon response [17, 52]. Coherently, we found IFNB1 gene expression induction in response to herring testes dsDNA transfection in both these cell lines (Supplementary Fig S5A). Additionally, both cell types had a significant number of siSETX cells with cGAS positive MN (Supplementary Fig S1H). In MCF-7 and MG-63 a siRNA against a control sequence was unable to induce a significant transcription of IFNB1, ISG15 and CXCL10 in our experimental conditions, while the three transcripts were significantly induced by SETX silencing (Fig. 5B and Supplementary Fig S5B). To correlate IFNB1 increased transcription with cGAS activation we pre-treated cells with the cGAS inhibitor RU.521 that indeed prevented IFNB1 transcription in siSETX cells (Fig. 5C). Notably, as observed in a long-term proliferation assay, the reduced growth of siSETX MG-63 cells is completely rescued by cGAS inhibition, (Fig. 5D), suggesting that unscheduled inflammation activation contributes to this defect. Since EXO1 silencing suppressed MN production in siSETX cells, we expected that it could also decrease IFNB1 induction. A positive result would have also implied that cGAS-positive MN contribute to activate downstream events in the inflammatory response in siSETX cells. Although EXO1 depletion induced per se IFNB1 transcription at least in MG-63 cells (Fig. 5E), it reduced the IFNB1 accumulation due to SETX silencing in both MG-63 (Fig. 5E) and MCF-7 cells (Supplementary Fig S5C). This further confirms the EXO1 involvement in the activation of innate immune response pathway caused by Senataxin deficiency and linked to MN formation. Consistently with previous findings [24], we found that also XPG depletion impairs the activation of interferon genes in SETX-deficient cells (Fig. 5F), although it has a minor impact on cGAS-dependent MN formation (Fig. 4G). However, we could not define the contribution of each gene in our experimental conditions since both EXO1 (Fig. 5E) and even more XPG silencing induce IFNB1 transcription (Fig. 5F). Thus, two different pathways of cGAS activation, depending on EXO1 or XPG, converge on a final step to trigger inflammation in SETX-deficient cells.

The cGAS dependent induction of interferon I detectable as IFNB1, ISG15 and CXCL10 transcriptional accumulation was also suppressed by silencing SPT4 in siSETX cells (Fig. 5G and Supplementary S5D). Finally, similarly to cGAS inhibition, cells silenced for both siSETX and siSPT4 are healthier than siSETX alone, as shown by cell viability assay (Fig. 5H).

Overall, these data suggest that, in the absence of Senataxin, R-loop-dependent cGAS positive MN accumulation contributes to induce an innate immune response, resulting in the inhibition of cell growth.

### High levels of EXO1 combined with a reduced expression of SETX leads to a poor prognosis in a subset of cancers

**Fig. 6:**
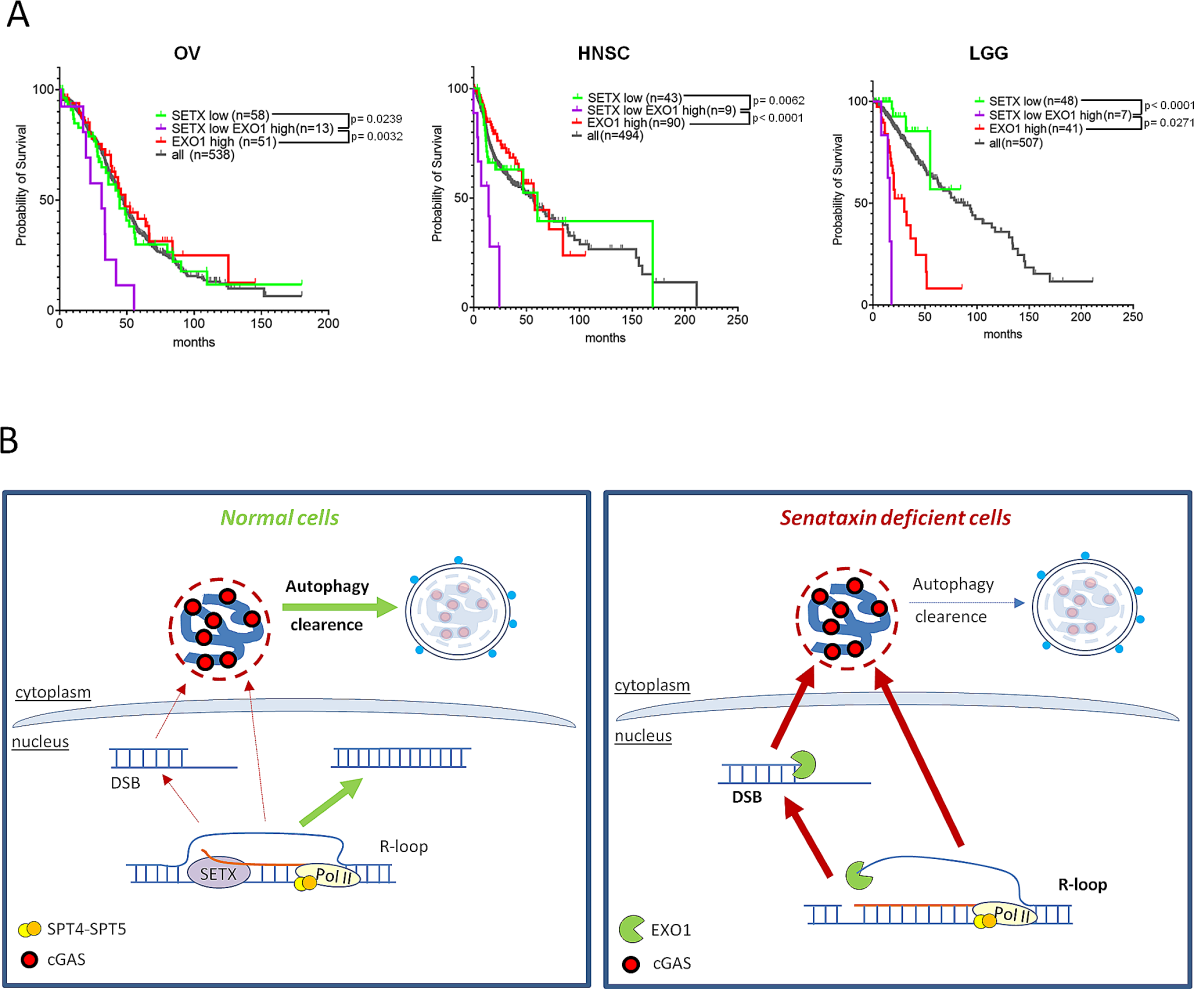
Senataxin and EXO1 interplay in cancer prognosis and model for R-loop dependent micronuclei accumulation and clearance in normal and Senataxin deficient cells. **(A)** Kaplan-Meier plot and log-rank t test values for the overall survival analysis of indicated cancer patients stratified for low/high expression of SETX and EXO1. OV = Ovarian serous cystadenocarcinoma; HNSC = Head and Neck squamous cell carcinoma; LGG = Brain Lower Grade Glioma. **(B)** Model for R-loop-dependent MN formation and clearance in normal and Senataxin-deficient cells. In normal cells, physiological R-loops are mainly resolved by SETX helicase, rarely resulting into DNA breaks. Eventually, chromatin fragments deriving from DNA breaks are included inside ruptured and cGAS-enriched MN and degraded in the cytoplasm by the autophagic process. In SETX-deficient cells, R-loops endure and consequently evolve more frequently toward unrepairable DNA breaks due to the activity of nucleases like EXO1, that cuts the single strand DNA portion and/or promotes an unscheduled long-range resection. The chromatin fragments produced are released into the cytoplasm inside MN that are mainly characterized by a discontinuous membrane, permissive for cGAS enrichment on DNA. The simultaneous presence of a defect in autophagy allows MN to escape degradation and to engage an interferon I response

To assess whether our findings about Senataxin and EXO1 roles in cGAS positive MN production could influence the pathogenesis of human cancer, we studied the expression of these two proteins in different publicly available data sets in the TCGA repository. In ovarian cancer (OV), head and neck squamous (HNSC) cell carcinoma and low-grade glioma (LGG), low levels of SETX expression did not correlate with a poorer prognosis in the tested cohorts (Fig. 6A). However, patients with simultaneously low levels of SETX and high levels of EXO1 had a significantly reduced survival probability when compared with the whole dataset, or the two sub-groups showing single alterations in SETX or EXO1 transcription level (Fig. 6A). Thus, an excessive engagement of the EXO1 nuclease activity in Senataxin deficient cells could result in a poorer prognosis to which R-loop-driven inflammation could contribute.

## Discussion

Here we found that the depletion of the RNA/DNA helicase Senataxin in human cells induces the release of MN caused by R-loop accumulation during cell cycle progression. Such MN include chromosome fragments deriving from R-loop-driven DNA damage that is a well-known hallmark of cells lacking Senataxin or its budding yeast ortholog Sen1 [1, 53].

We also found that in Senataxin deficient cells, the MN elicited in absence of any stress condition are prone to be bound by the cGAS protein, the apical sensor of innate immunity [54]. On the contrary, DSBs induced by a genotoxic treatment (NCS) or due to defective DNA break repair (BRCA1 KD) produce high amount of MN but, in proportion, they are rarely cGAS positive. Investigating the reasons for this massive and frequent recruitment of cGAS inside MN, we identified in Senataxin deficient cells the recurring presence of a discontinuous micronuclear membrane that allows the shuttling of highly mobile proteins. We also found that the clearance of such permeable MN is favoured by autophagy and that this process is impaired in Senataxin-deficient cells. Particularly we found that both p62/SQSTM1, an autophagy receptor and cGAS-binding protein, and LC3, a protein involved in autophagy substrate binding and autophagosome biogenesis are reduced in absence of SETX. Additionally, while MN with a broken membrane are marked by p62/SQSTM1 both in control and SETX depleted cells, LC3 recruitment is impaired in the latter. Coherently a micronucleophagic pathway involving both p62/SQSTM1 and LC3 was previously described [18, 32, 38] and autophagy defects were detected in AOA2 deriving cells [39]. We also found that the residual MN production in SETX deficient cells was dependent on the activity of EXO1, an exonuclease that catalyzes long range removal of nucleotides during DSB repair or stalled replication fork processing with well-known consequences on genome instability when its nucleolytic activity is excessively engaged [55]. Moreover, in budding yeast, Exo1 was shown to process unprotected replication forks arrested at R-loop-prone sites of collisions with transcription [56]. While EXO1 resection activity could be engaged at different stages of the process that leads from DNA damage formation to exposure of DNA in the cytoplasm, one possibility is that EXO1 directly processes R-loops. According with its biochemical properties, EXO1 could indeed cut 5’ flap DNA structures [47] generated at broken R-loops (Fig. 6B). However, in both control and Senataxin-depleted cells, micronuclear chromatin is γ-H2AX-positive, a marker associated with the activation of a DNA damage response [41]. Overall, our data suggest that, in SETX-deficient cells, MN formation is related to the R-loop dependent induction of DNA damage, while cGAS accumulation is promoted by both R-loop processing and the impairment of the autophagic process (Fig. 6B).

Recent studies have linked the unrestrained accumulation of nuclear R-loops with cGAS-dependent innate immune response [22–24]. More specifically, in cells lacking Senataxin or similarly BRCA1, it has been shown that RNA:DNA hybrids, once excised from nuclear R-loops by the XPG/XPF endonucleases, are released into the cytoplasm where they directly bind cGAS and activate the inflammatory response [24]. The release of such RNA:DNA hybrids in the cytoplasm is not restricted to cycling cells and occurs via an active nuclear export mechanism [24]. Thus, the pathway of R-loop-dependent cGAS activation that we unveil here is distinct from the one above in multiple aspects, since it occurs (i) via micronuclei formation, (ii) exclusively in cycling cells, (iii) relies mainly on EXO1 and (iv) it is more evident in SETX deficient than in BRCA1 defective cells, because it depends on R-loop-derived DNA damage rather than on impaired DSB repair by homologous recombination. However, a question arising from our data is whether RNA:DNA hybrids may also contribute to cGAS recruitment in MN. Indeed RNA:DNA hybrids were found in MN, where their processing contributes to the high instability characterizing the genomic material of those cytoplasmatic structures [57]. Although we cannot rule out that cGAS directly recognizes RNA:DNA hybrids in MN, this hypothesis hardly explains the massive cGAS recruitment on MN DNA observed in our immunofluorescence analysis. Moreover, the EXO1-dependent accumulation of phosphorylated RPA32 on S4/S8 in the nucleus of SETX-deficient cells could suggest that resected DNA is an intermediate between R-loop accumulation and MN formation. However, if RNA:DNA hybrids were not processed in the nucleus, they should be a minor portion of genomic material released into MN. Additionally, the maximal activation of cGAS requires a long dsDNA molecule [58, 59] and cGAS-mediated IFNB induction is at least one order of magnitude more in presence of dsDNA than RNA:DNA hybrids [60]. Thus, we favour the hypothesis that RNA:DNA hybrids could have, if any, an indirect role in the process described here, acting, for instance, as nucleation sites for specific chromatin changes that are known to affect cGAS recruitment at MN DNA [31]. The peculiar molecular features influencing cGAS recruitment in Senataxin-dependent MN constitute a relevant aspect that will require further investigations. However, the existence of different pathways through which the processing of persistent nuclear R-loops leads to innate immune response activation is in line with the notion that this process contributes to multiple pathological contexts, including cancer, neurodegenerative diseases and autoimmune disorders, and therefore should be strictly regulated.

In accordance with previous observations in other human cell lines [24], we found that cancer cells depleted of Senataxin activate interferon I and interferon-stimulated genes transcription in a cGAS-dependent manner. Notably, in our context, the formation of inflamed MN and the downstream activation of the interferon genes is dependent on EXO1 activity, indicating a link between these two events. The presence of a MN-dependent activation of interferon expression was further demonstrated by the translocation of IRF3 inside the nucleus of MN positive SETX depleted cells. Since in SETX/EXO1 double KD a partial interferon induction is retained and XPG contributes as well to such activation (Fig. 5E-F) [24], our data are coherent with the fact that at least two different R-loop processing pathways concur to trigger interferon upregulation. The interferon response could reduce cell growth, as we found here in SETX-depleted cells, and, in a more systemic context, it could induce inflammation. Therefore, our data suggest that cells defective for Senataxin are characterized by a mild genomic instability and low levels of MN release compared to control cells, but, since these MN engage cGAS, they contribute to activate the innate immunity and a low-grade chronic inflammation even in the absence of external genotoxic events. This possibility is of particular interest since Senataxin protein is reduced in a subset of cancer types and the relationship between inflammation and cancer development, progression and therapy is of great relevance, although multifaceted [61]. Coherently with the role of EXO1 in promoting cGAS positive MN in a SETX deficient background, we found that in ovarian cancer, head and neck squamous cell carcinoma and glioma the concurrent presence of low SETX and high EXO1 expression levels leads to poor survival. This is particularly relevant since alterations of EXO1 expression could enhance and uncover a neglected detrimental effect of SETX deficiency in cancer.

On the other side, in Senataxin associated syndromes, like AOA2 or ALS4, R-loops accumulating in cycling cells in the brain [62], like glia, could start a low and chronic level of inflammation that contribute to neurodegeneration. A link between genomic instability and inflammation was effectively found in Ataxia Telangiectasia [63, 64], a disease characterized by ataxia, like AOA2. Considering these findings and our data, a low level of inflammation deriving from genome instability and self-DNA release in the cytoplasm could be a common trait of ataxias. In the case of Senataxin ablation, these events derive all from R-loops accumulation and could be, at least in part, mediated by MN production as demonstrated by the reduction of IFNB1 expression after EXO1 depletion. The relationship between R-loops and inflammation is corroborated by the aetiology of AGS, a neuroinflammatory disease mainly caused by mutations in the R-loop removing enzyme RNase H [18, 20, 21]. Finally, our data about the accumulation of MN with a ruptured membrane in Senataxin depleted cells, could contribute to explain a recently reported case of SLA characterized by co-inheritance of variants in SETX, FUS and LMNA [65].

As tumor cells must cope with replication stress for their survival and R-loops greatly contribute to replication stress, factors that limit R-loop formation could be promising novel anticancer drug targets. On the other side, the possibility to reduce R-loop accumulation in cells with alteration of Senataxin activity, or as the result of other genetic diseases due to the loss of anti-RNA:DNA hybrid factors, is also of great interest. Since R-loops are essentially co-transcriptional, a possibility to rescue R-loop homeostasis defects is to modulate RNA polymerase processivity (Fig. 6B). Previous studies found SPT4 gene to act as a suppressor of R-loop-driven replication-transcription collisions and DNA damage in cells lacking Sen1, the yeast ortholog of Senataxin [33]. SPT4, together with SPT5, forms the evolutionarily conserved DSIF complex that regulates RNA polymerase elongation stage [36] and pausing at transcriptional initiation and termination [66, 67], all events that are strictly correlated with R-loop formation [5]. Consistent with yeast data, here we found that a down-regulation of SPT4, while leaving unaffected cell growth, at least in a short time, reduces R-loop accumulation in Senataxin depleted cells and, as consequence, MN accumulation, cGAS recruitment and interferon transcription. A long-term assay also demonstrates the ability of siSPT4 to increase siSETX cell growth, despite a cell line-specific toxicity associated with the knock-down of this protein. Thus, at least in theory, a targeted inhibition of SPT4 could be used as strategy to buffer R-loop-dependent detrimental effects in neurological disorders caused by Senataxin loss. It is noteworthy that the inhibition of SPT4 in human cells and model organisms has been proposed as a strategy to reduce aberrant transcription across repeat expansions in certain neurological diseases, including the case of R-loop-prone *C9orf72* gene expanded in ALS [68, 69]. While it is debated whether SPT4 ablation selectively reduces transcription at expanded disease loci [68] or more globally [70], our observations, indicating that Senataxin defects can be rescued by modulating transcription elongation, warrant future investigations on this direction.

### Electronic supplementary material

Below is the link to the electronic supplementary material.

Supplementary Material 1

## Data Availability

The data that support the findings of this study are available from the corresponding authors upon reasonable request.
