# Supplementary material for: R-loops and impaired autophagy trigger cGAS-dependent inflammation via micronuclei formation in Senataxin-deficient cells
Source: Cell Mol Life Sci. 2024 Aug 9;81(1):339. doi: 10.1007/s00018-024-05380-3 (PMC11335261; doi:10.1007/s00018-024-05380-3)
Supplement: Supplementary file 1 — Supplementary Material 1 [file 18_2024_5380_MOESM1_ESM.pdf]

R-loops and impaired autophagy trigger cGAS-dependent inflammation via micronuclei formation in Senataxin-deficient cells. Laura Zannini, Miriana Cardano, Giordano Liberi and Giacomo Buscemi

Fig S1

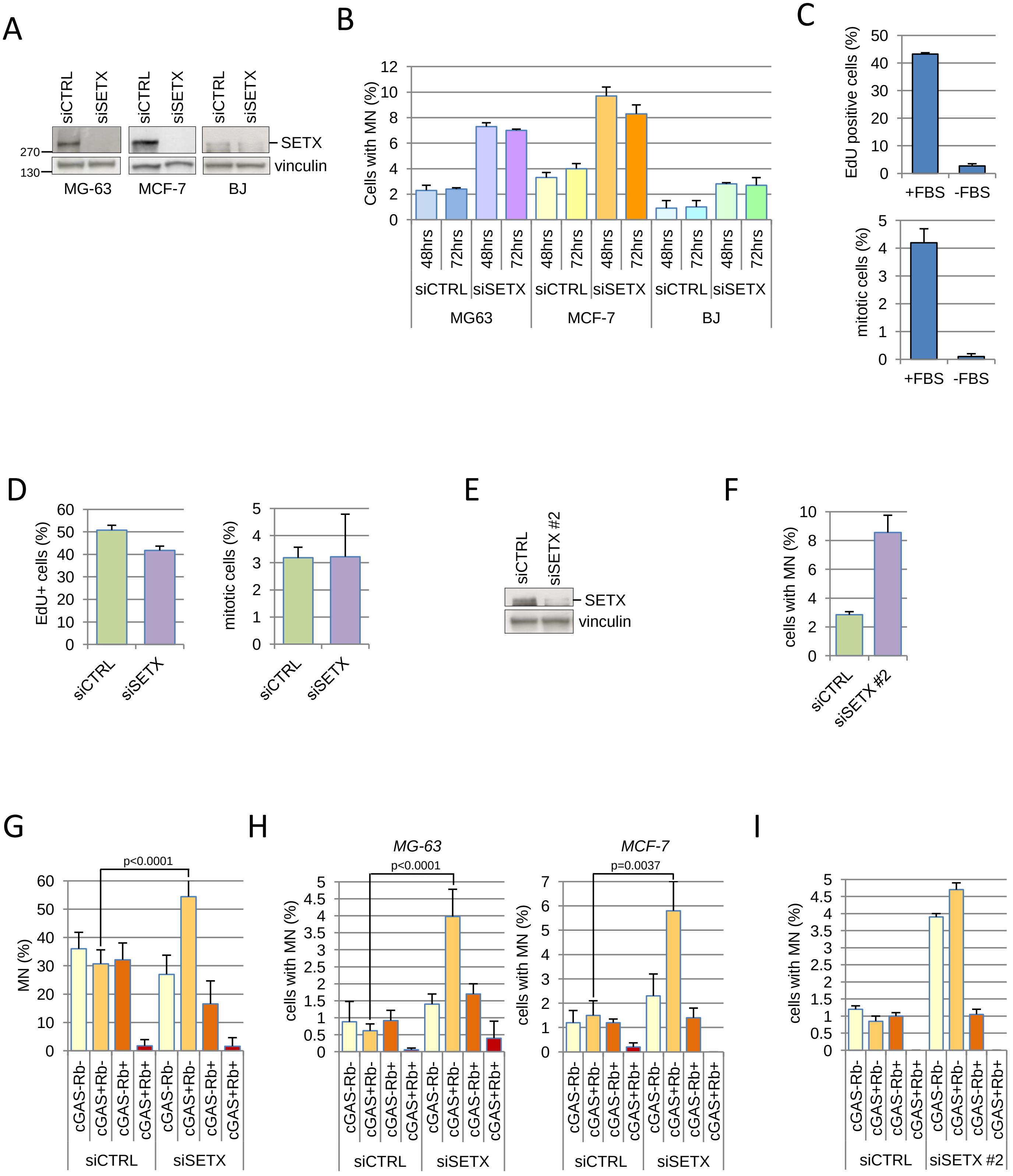

Fig S1

J

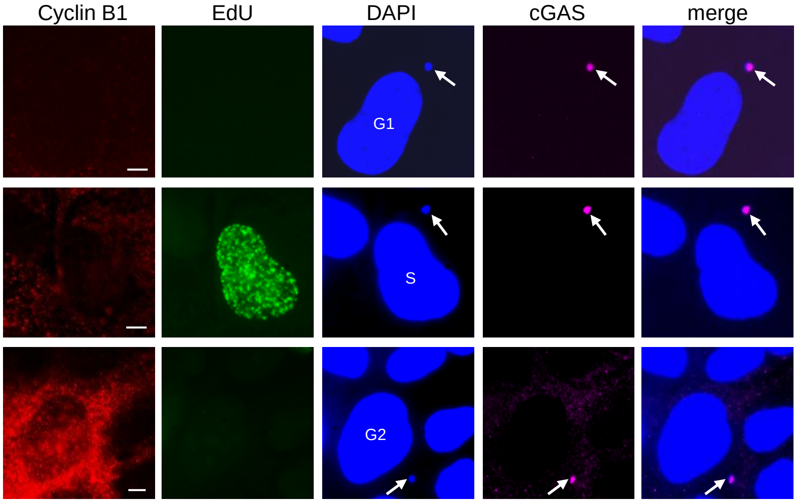

Cyclin B1
EdU
DAPI
cGAS
merge
G1
S
G2

K

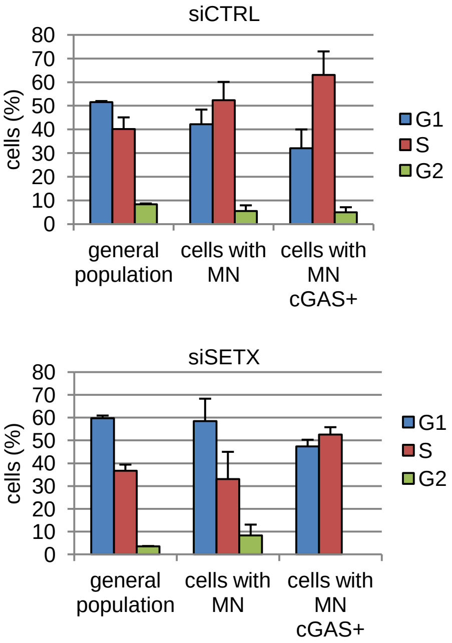

siCTRL
cells (%)
80
70
60
50
40
30
20
10
0
general population
cells with MN
cells with MN cGAS+
G1
S
G2
siSETX
cells (%)
80
70
60
50
40
30
20
10
0
general population
cells with MN
cells with MN cGAS+
G1
S
G2

Fig S2

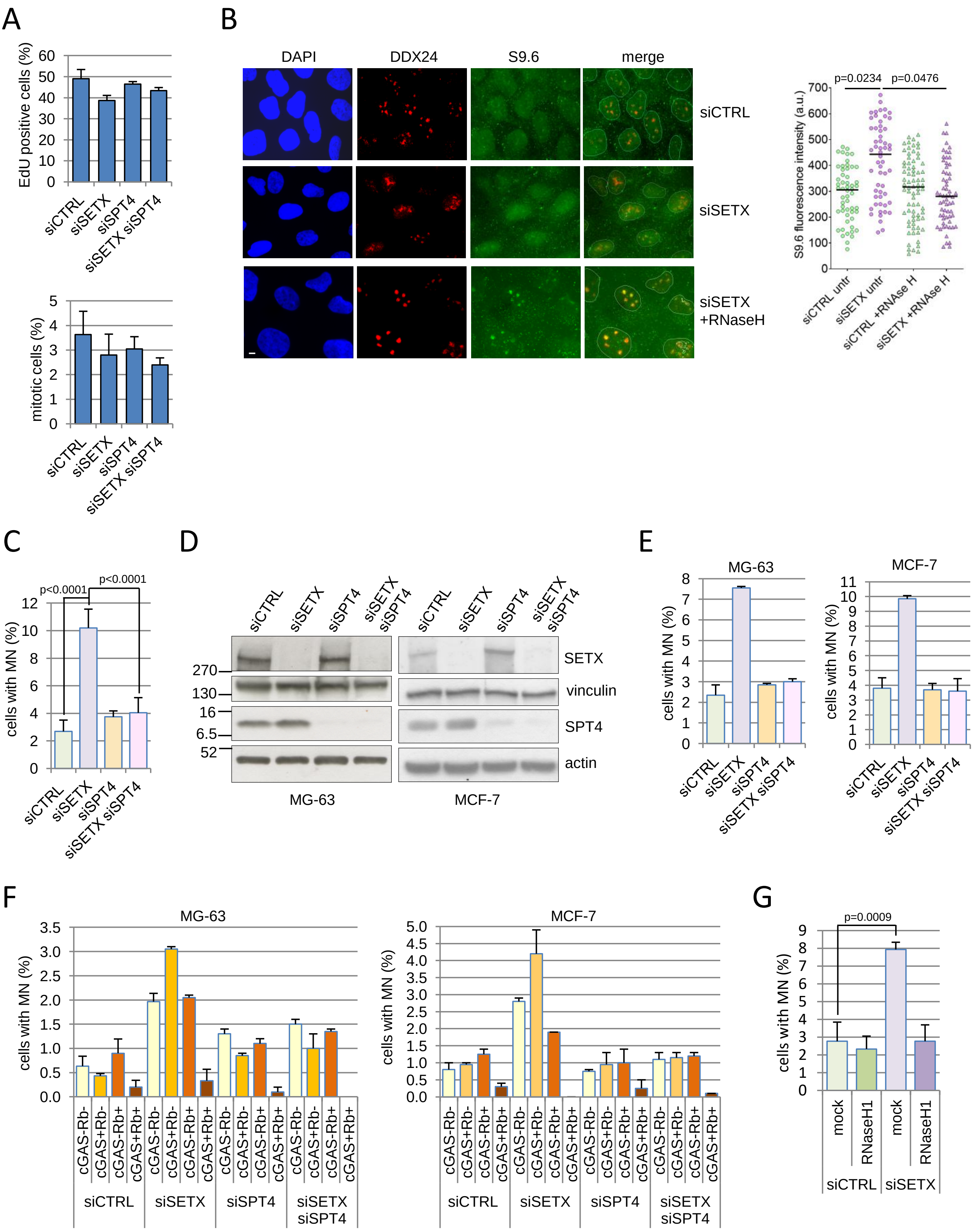
A
EdU positive cells (%)
siCTRL
siSETX
siSPT4
siSETX siSPT4
mitotic cells (%)
B
DAPI
DDX24
S9.6
merge
siCTRL
siSETX
siSETX +RNaseH
p=0.0234
p=0.0476
S9.6 fluorescence intensity (a.u.)
siCTRL untr
siSETX untr
siCTRL +RNAse H
siSETX +RNAse H
C
p<0.0001
p<0.0001
cells with MN (%)
D
SETX
vinculin
SPT4
actin
MG-63
MCF-7
E
MG-63
MCF-7
cells with MN (%)
F
MG-63
MCF-7
cells with MN (%)
cGAS-Rb-
cGAS+Rb-
cGAS-Rb+
cGAS+Rb+
siCTRL
siSETX
siSPT4
siSETX siSPT4
G
p=0.0009
cells with MN (%)
mock
RNaseH1
siCTRL
siSETX

Figure S3

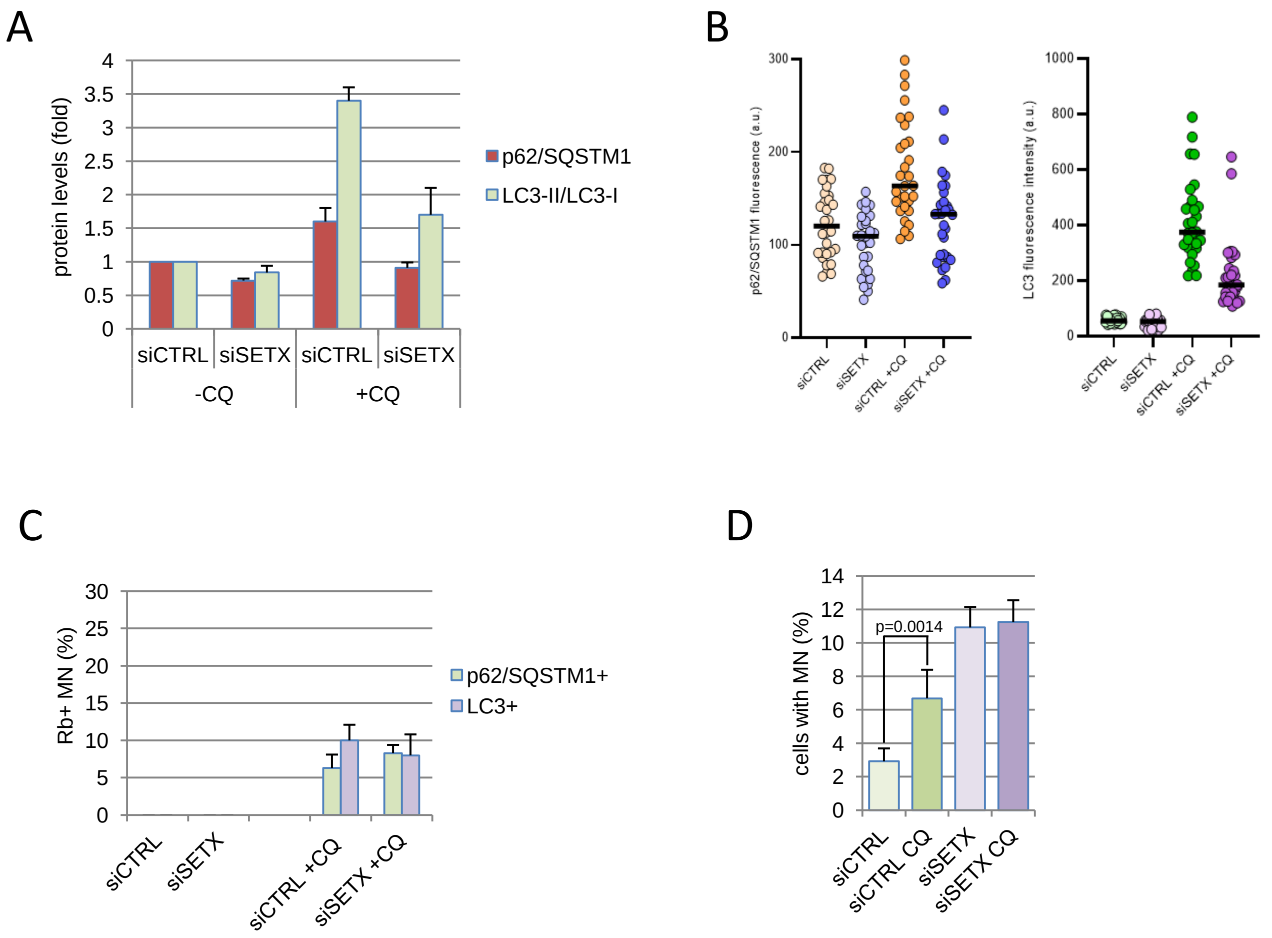
A
protein levels (fold)
4
3.5
3
2.5
2
1.5
1
0.5
0
siCTRL
siSETX
siCTRL
siSETX
-CQ
+CQ
p62/SQSTM1
LC3-II/LC3-I
B
p62/SQSTM1 fluorescence (a.u.)
300
200
100
0
siCTRL
siSETX
siCTRL +CQ
siSETX +CQ
LC3 fluorescence intensity (a.u.)
1000
800
600
400
200
0
siCTRL
siSETX
siCTRL +CQ
siSETX +CQ
C
Rb+ MN (%)
30
25
20
15
10
5
0
siCTRL
siSETX
siCTRL +CQ
siSETX +CQ
p62/SQSTM1+
LC3+
D
cells with MN (%)
14
12
10
8
6
4
2
0
p=0.0014
siCTRL
siCTRL CQ
siSETX
siSETX CQ

Fig S4

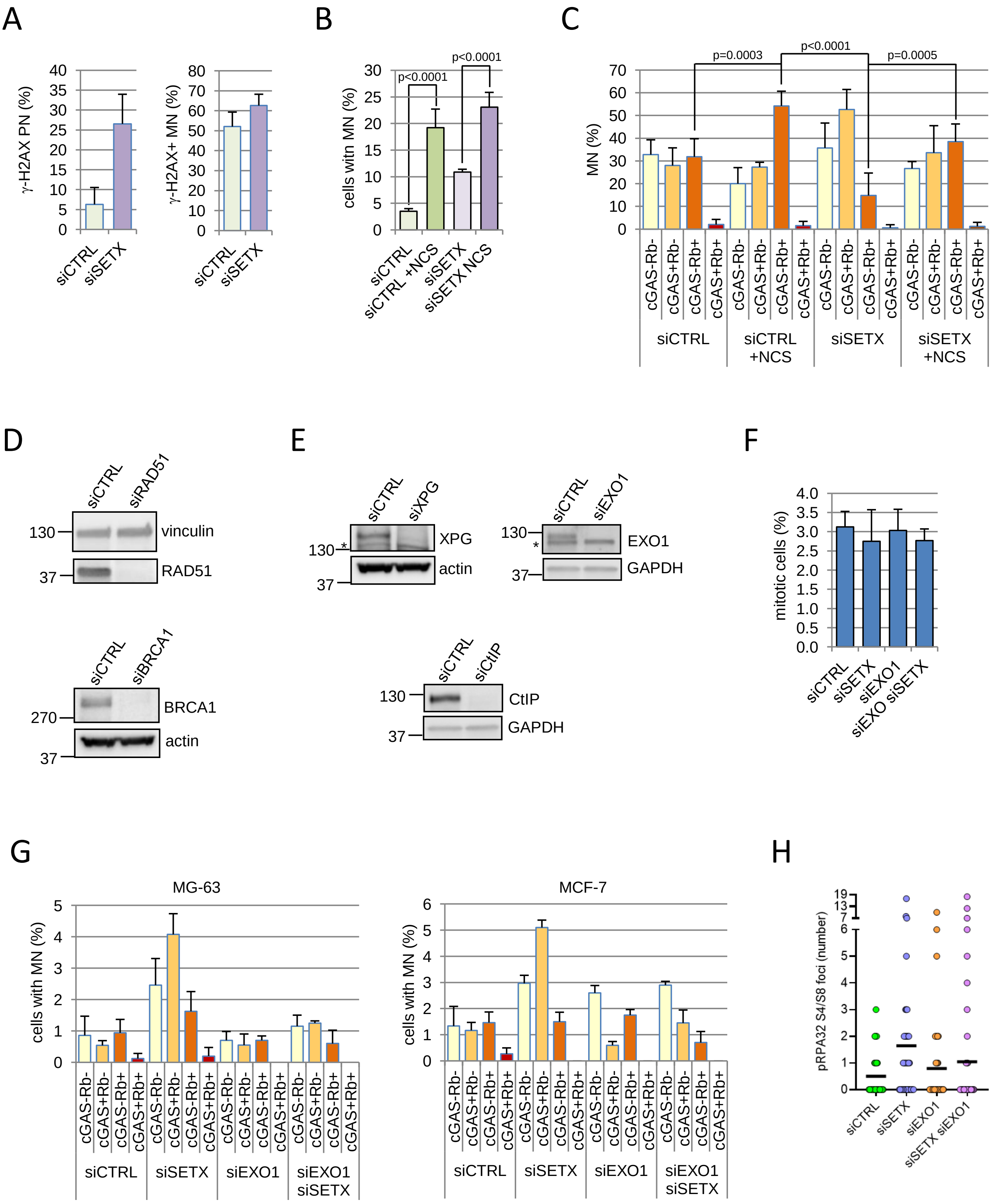
A
γ-H2AX PN (%)
γ-H2AX+ MN (%)
siCTRL
siSETX
B
cells with MN (%)
p<0.0001
p<0.0001
siCTRL
siCTRL +NCS
siSETX
siSETX NCS
C
MN (%)
p=0.0003
p<0.0001
p=0.0005
cGAS-Rb-
cGAS+Rb-
cGAS-Rb+
cGAS+Rb+
siCTRL
siCTRL +NCS
siSETX
siSETX +NCS
D
siCTRL
siRAD51
vinculin
RAD51
siBRCA1
BRCA1
actin
E
siXPG
XPG
siEXO1
EXO1
GAPDH
siCtIP
CtIP
F
mitotic cells (%)
siEXO1
siEXO siSETX
G
MG-63
MCF-7
cells with MN (%)
siEXO1 siSETX
H
pRPA32 S4/S8 foci (number)
siSETX siEXO1

Fig S5

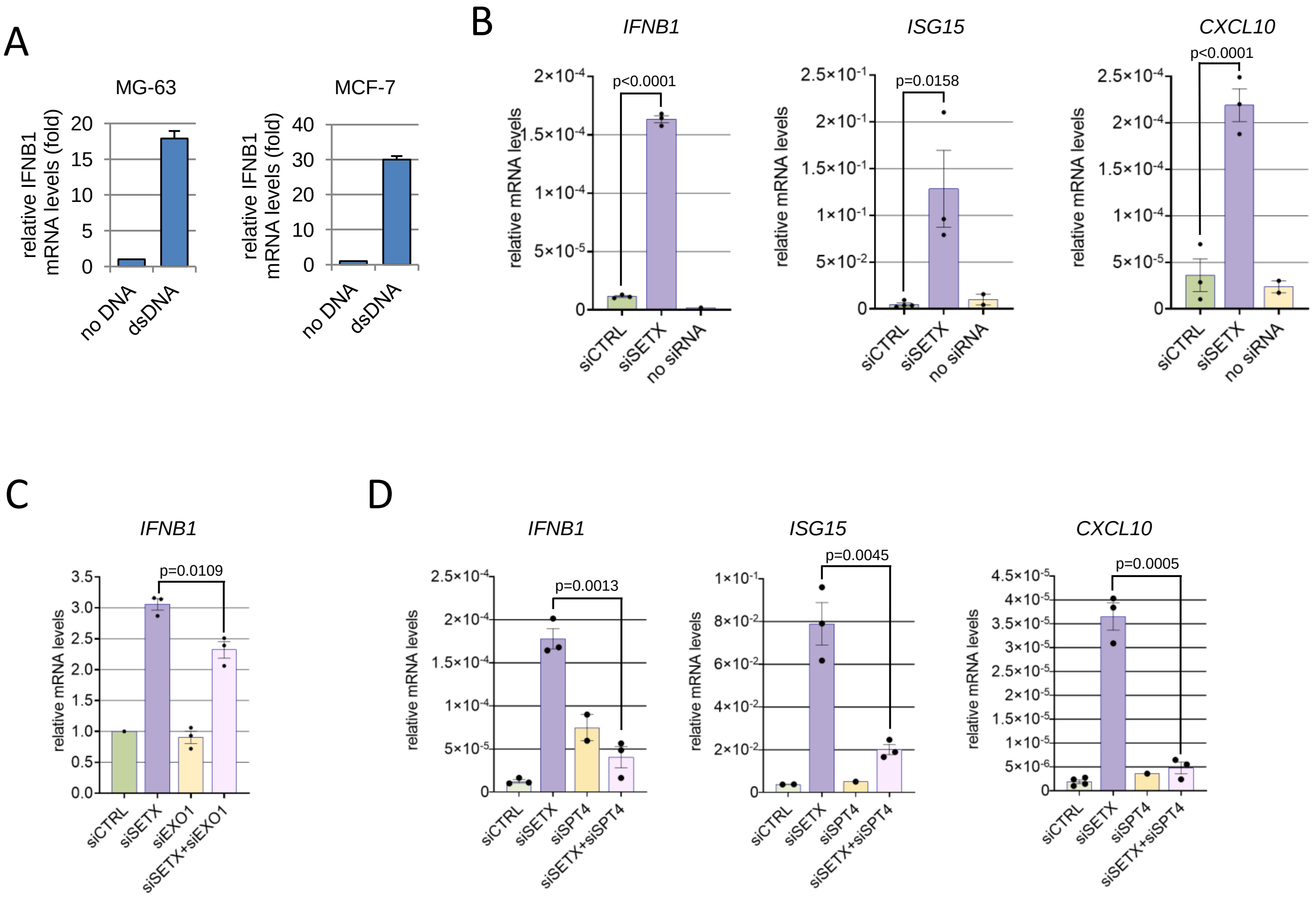

A
MG-63
MCF-7
relative IFNB1 mRNA levels (fold)
no DNA
dsDNA
B
IFNB1
ISG15
CXCL10
relative mRNA levels
p<0.0001
p=0.0158
siCTRL
siSETX
no siRNA
C
p=0.0109
siEXO1
siSETX+siEXO1
D
p=0.0013
p=0.0045
p=0.0005
siSPT4
siSETX+siSPT4

R-loops and impaired autophagy trigger cGAS-dependent inflammation via micronuclei formation in Senataxin-deficient cells. Laura Zannini, Miriana Cardano, Giordano Liberi and Giacomo Buscemi

SUPPLEMENTARY FIGURES LEGENDS

Supplementary Figure 1. **(A)** MG-63, MCF-7 and BJ-hTERT cells were transfected with a control (siCTRL) or SETX siRNA (siSETX) and Senataxin protein levels assessed by western blot at 48 hrs after transfection. Vinculin was used as loading control. **(B)** MG-63, MCF-7 and BJ-hTERT cells silenced for CTRL or SETX were fixed after 48 or 72hrs and stained with DAPI. Cells with a micronucleus were enumerated scoring at least 500 nuclei, the graph shows the mean and s.d. of three biologically independent experiments. **(C)** MG-63 cells grown for 48hrs in presence (+FBS) or in absence of serum (-FBS) were marked with EdU to identify EdU-positive replicating cells (top) or fixed and stained with DAPI to identify mitotic figures (bottom). The graph shows the mean and s.d. of two biologically independent experiments. **(D)** Left: U2OS transfected with control or SETX siRNAs after 48hrs were marked with EdU (left) and stained to reveal replicating cells (at least 500 nuclei were scored). The same cells were also fixed and stained with DAPI (right) to identify Mitotic figures (at least 1000 nuclei were scored). The graph shows the mean and s.d. of three biologically independent experiments. (**E)** U2OS cells silenced using an alternative siRNA for SETX were tested for SETX protein content. Vinculin was used as a loading control. **(F)** Cells as in (E) were analyzed for MN presence by DAPI staining. MN were enumerated, scoring at least 500 nuclei, as described in Figure 1. The graph shows the mean and s.d. of two biologically independent experiments. **(G)** U2OS cells transfected with control or SETX siRNA after 48hrs were fixed and immunostained with primary anti-cGAS or Rb antibodies. The graph shows the percentage of MN negative for the presence of both proteins (cGAS- Rb-) positive for one (cGAS+ Rb- or cGAS- Rb+) or both (cGAS+ Rb+) proteins. Data were obtained scoring at least 50 MN and performing at least five biologically independent experiments. The graph shows the mean and s.d. of each category and the indicated exact p value was obtained comparing cGAS+Rb- subtype frequency in siCTRL and siSETX samples, using the two-tailed unpaired Student's t test. **(H)** MG-63 (left) and MCF-7 (right) cells were silenced as in (A), fixed and stained. Graphs show data obtained by immunostaining cells silenced or not for SETX. Bars represent the percentage of cells positive for cGAS and/or Rb (see G). Data were obtained scoring at least 500 nuclei, graph is representative of three independent experiments. The bar shows the mean frequency and s.d. of each category. The exact p value was obtained comparing data for cGAS+Rb- category, using the two-tailed unpaired Student's t test. **(I)** U2OS cells were analysed as in (G) using an alternative siRNA for SETX gene. Data were obtained scoring at least 50 MN and performing two biologically independent experiments. **(J)** Representative images of EdU treated cells (10μM, 45 minutes), fixed, stained for EdU and

immunostained with anti-cyclin B1 and anti-cGAS antibodies. Cells double negative for cyclin B1 and EdU were considered as in G1 phase, Edu positive in S phase, cyclin B1 positive in G2. DAPI was used to counterstain nuclei and exclude mitotic cells. White bar = 5µm **(K)** General population (all cells), cells with at least one MN, and cells with a cGAS positive MN were evaluated for cell cycle phase as shown in (J). Bars represent the fraction of cells in a specific cell cycle stage in siCTRL (top) and siSETX samples (bottom). The graphs show the mean and s.d. of two biologically independent experiments.

Supplementary Figure 2. **(A)** U2OS transfected with the indicated siRNAs were marked with EdU to identify EdU-positive replicating cells (top) or fixed and stained with DAPI to identify mitotic figures (bottom). The graphs show the mean and s.d. of two biologically independent experiments. **(B)** U2OS cells transfected with control (CTRL) or SETX siRNA after 48hrs were fixed, treated with recombinant RNaseH and stained by immunofluorescence with the S9.6 antibody against DNA/RNA hybrids (green) and DDX24 (red) used a marker of nucleoli. DAPI (blue) was used to mark nuclei. Representative images are shown (left). White bar = 5µm**.** Pictures were acquired for each sample and the intensity of the green signal in the nucleus excluding nucleoli regions (red signal) was quantified (arbitrary unit) for 30 cells. Results from three biologically independent experiments are shown in the graph (right), including the median and the specific exact p values obtained using the one way ANOVA plus Bonferroni post hoc test. **(C)** MN were analysed following silencing (same siRNAs as A). Data were obtained scoring at least 50 MN in each of four biologically independent experiments. The exact p value was obtained using the two-tailed unpaired Student's t-test. **(D)** Western blot analysis of MG-63 (left panels) and MCF-7 (right panels) cells transfected with control (siCTRL) or siSETX, siSPT4 and siSETX+siSPT4 siRNAs. Senataxin and SPT4 levels were assessed 72hrs after transfection. Vinculin and actin were used as loading controls. **(E)** MG-63 and MCF-7 cells were transfected to silence the indicated genes and tested for MN accumulation by DAPI staining. The values in the graph are mean±s.d. of two biologically independent experiments. **(F)** MG-63 (left) or MCF-7 (right) cells transfected with the indicated siRNAs were fixed and immunostained with primary anti-cGAS and Rb antibodies. The graph shows the percentage (mean±s.d.) of cells with MN, resulting positive or negative for the presence of cGAS and/or Rb. The values in the graph are mean±s.d and data were obtained by scoring at least 50 MN in each of at least three biologically independent experiments. **(G)** Graph showing the percentage of cells silenced for CTRL or SETX and positive to GFP or GFP-RNAseH1, containing a MN. At least 100 GFP positive nuclei were scored. Bars represent means with s.d. obtained from three biologically independent experiments. The exact p value was obtained using the two-tailed unpaired Student's t test; RNaseH1 samples are not statistically different.

Supplementary Figure 3. **(A)** Quantification of p62/SQSTM1 and LC-II/LC3-I ratio using normalized proteins level obtained by western blot analysis (see Fig 3A). Bars represent means with s.d. from two biologically independent experiments. **(B)** Cells silenced with siCTRL and siSETX were treated or not for 24hrs with 15μM CQ, fixed and assayed by immunofluorescence to evaluate p62/SQSTM1 and LC3 protein levels (see Fig 3B). The signal intensity of p62/SQSTM1 (left) and LC3 (right) in the cytoplasm (DAPI staining was used to exclude nuclear signals) in the cytoplasm was quantified and expressed in an arbitrary unit (a. u.) for each cell from two biologically independent experiments. The median of the data is included in the graph. **(C)** Cells silenced with siCTRL and siSETX were treated for 24hrs with 15μM CQ, fixed and assayed by immunofluorescence for p62/SQSTM1 and LC3 protein expression and localization (see Figure 3C). Bars (means with s.d.) represent the fraction of Rb positive MN marked by p62 (p62+) or LC3 (LC3+) puncta in siCTRL or siSETX cells treated or not with CQ. At least 30 MN per sample were evaluated in two biologically independent experiments. **(D)** MN were analysed by DAPI staining comparing siCTRL and siSETX cells exposed for 30hrs to 15μM chloroquine (CQ). Data were obtained from four biologically independent experiments. The exact p value was obtained using the two-tailed unpaired Student's t test; siSETX and siSETX+CQ samples are not statistically different.

Supplementary Figure 4. **(A)** siCTRL and siSETX cells were immunostained with anti-γ-H2AX as described in Figure 4A and B. Nuclei (primary nuclei, PN) and micronuclei (MN) were stained with DAPI. Cells with a nucleus containing more than 5 γ-H2AX foci were considered as positive; only cells without MN were included in this group. MN containing one or more γ-H2AX foci were considered as positive The graphs show the frequencies of positive PN (left panel; mean and s.d) and MN (right panel; mean and s.d.). 50 PN and 30 MN were evaluated for three biologically independent experiments. **(B)** U2OS cells transfected with control or SETX siRNA after 24hrs were exposed to 16nM neocarzinostatin (NCS), successively fixed and stained with DAPI to score cells with MN. Bars represent means with s.d. obtained from three biologically independent experiments and the exact p value was obtained using the two-tailed unpaired Student's t test. **(C)** Cells treated as in (B) were immunostained with primary anti-cGAS and Rb antibodies. Data were obtained scoring at least 50 MN for each of four experiments. Bars represent means with s.d. and the exact p value was obtained using the one way ANOVA plus Bonferroni post hoc test. **(D)** U2OS cells were transfected twice with control (CTRL), RAD51 or BRCA1 siRNAs and tested by western blot for RAD51 (upper panel) and BRCA1 (lower panel) protein downregulation. Actin was used as loading control. **(E)** U2OS cells silenced with the indicated siRNAs were lysed and protein samples analysed by western blot using specific antibodies. GAPDH and actin were used as loading controls. **(F)** U2OS transfected with the indicated siRNAs were fixed and stained with DAPI to enumerate mitotic figures. Bars represent means with s.d. of two biologically independent experiments. (**G)** MG-63 and

MCF-7 cells transfected with the indicated siRNAs were immunostained as described in (C). Bars represent means with s.d. obtained from two biologically independent experiments. **(H)** pRPA32 S4/S8 bright foci number evaluated by immunofluorescence staining for each of 30 cells and two biologically independent experiments. Means are represented.

Supplementary Figure 5. **(A)** MG-63 (left) and MCF-7 (right) cells were transfected with dsDNA and mRNA was extracted from cells 24hrs later. Samples were tested by RT-qPCR to analyse IFNB1 mRNA expression levels (relative to GAPDH). Data in the graph (means±s.d.) are representative of two biologically independent experiments. **(B)** Analysis, by RT-qPCR, of IFNB1, ISG15 and CXCL10 mRNAs expression levels (relative to GAPDH) in MCF-7 cells transfected with siCTRL, siSETX or with no siRNA. mRNA was extracted from cells 48hrs after a second round of silencing. The values in the graphs are mean±s.e.m. of three biologically independent experiments. The exact p values were obtained using the two-tailed unpaired Student's t test. (**C**) Analysis, by RT-qPCR, of IFNB1 mRNA expression levels (relative to GAPDH) in MCF-7 cells pre-silenced with EXO1 siRNA and successively silenced for CTRL or SETX. The values in the graph are mean±s.e.m. of three biologically independent experiments and the exact p value was obtained using the two-tailed unpaired Student's t test. **(D)** RT-qPCR analysis of IFNB1, ISG15 and CXCL10 mRNAs expression levels (relative to GAPDH) in MCF-7 cells transfected with siCTRL, siSETX, siSPT4 or siSETX+siSPT4, as described in (B). The values in the graphs are mean±s.e.m. of three biologically independent experiments. The exact p values were obtained using the two-tailed unpaired Student's t test.

NAME	COMPANY	CATALOG No.	REFERENCE
siRNAs			
SETX	Eurofins	GCCAGAUCGUAUACAAUUA	Richard et al., 2021
SETX #2	Qiagen	FlexiTube siRNA Hs_SETX_2	
siSPT4	Qiagen	FlexiTube siRNA Hs_SUPT4H1_5 and _6	
siBRCA1	Eurofins	CAGCUACCCUUCCAUCAUA	He et al., 2020
siCTIP	Eurofins	AAGCUAAAACAGGAACGAAUC	Makharashvili et al., 2014
siXPG	Eurofins	GAACGCACCUGCUGCUGUA	Crossley et al., 2023
siEXO1	Eurofins	GCACGUAAUUCAAGUGAUG	Kim et al., 2021
siCTRL	Eurofins	CGUACGCGGAAUACUUCGA	
siCTRL	Qiagen	AllStars Negative Control siRNA	
siRAD51	Ambion	AM16708	
Primers			
IFNB1-for	Eurofins	ACGCCGCATTGACCATCTAT	
IFNB1-rev	Eurofins	TAGCCAGGAGGTTCTCAACA	
ISG15-for	Eurofins	GAGAGGCAGCGAACTCATCT	
ISG15-rev	Eurofins	CTTCAGCTCTGACACCGACA	
CXCL10-for	Eurofins	TGGCATTCAAGGAGTACCTA	
CXCL10-rev	Eurofins	TTGTAGCAATGATCTCAACACG	
GAPDH-for	Eurofins	ACCACAGTCCATGCCATCAC	
GAPDH-rev	Eurofins	TCCACCACCCTGTTGCTGTA	
Antibodies			
SETX rabbit polyclonal	Novusbio	NBP1-94712	
SPT4 rabbit monoclonal D3P2W	Cell Signaling Tech.	64828	
BRCA1 rabbit polyclonal	Sigma-Aldrich	07-434	
CTIP mouse monoclonal D-4	Santa Cruz Biotech.	27339	
XPG rabbit polyclonal	Sigma-Aldrich	X1629	
EXO1 rabbit polyclonal	Genetex	GTX109891	
Actin mouse monoclonal	Sigma-Aldrich	A2066	
Vinculin mouse monoc. hVIN-1	Sigma-Aldrich	V9131	
GAPDH monoclonal mouse	Sigma-Aldrich	SAB1405848	
cGAS rabbit monoclonal D1D3G	Cell Signaling Tech.	15102	
Rb mouse monoclonal 4H1	Cell Signaling Tech.	9309	
γ-H2AX pSer139 mouse monoclonal JBW301	Sigma-Aldrich	05-636	
DDX24 rabbit polyclonal	Bethyl Laboratories	A300-696	
Cyclin B1 mouse monocl. (GNS-1)	BD-Pharmingen	554176	
Cyclin A rabbit polyclonal	Santa Cruz Biotech.	H-432	
S9.6 mouse monoclonal	-	purified from hybridoma HB-8730	
LC3 rabbit monoclonal (D11)	Cell Signaling Tech.	3868	
pRPA32 S4/S8 rabbit polyclonal	Bethyl Laboratories	A300-245	
RAD51 mouse monoclonal (14B4)	GeneTex	GTX70230	
p62/SQSTM1 rabbit polyclonal	GeneTex	GTX100685	
GFP mouse monoclonal 3E6	Thermo Fisher Sc.	A-11120	

Supplementary Table 1.

Richard P, Feng S, Tsai YL, Li W, Rinchetti P, Muhith U, Irizarry-Cole J, Stolz K, Sanz LA, Hartono S, Hoque M, Tadesse S, Seitz H, Lotti F, Hirano M, Chédin F, Tian B, Manley JL. (2021) SETX (senataxin), the helicase mutated in AOA2 and ALS4, functions in autophagy regulation. Autophagy. 17(8):1889-1906.

He YJ, Meghani K, Caron MC, Yang C, Ronato DA, Bian J, Sharma A, Moore J, Niraj J, Detappe A, Doench JG, Legube G, Root DE, D'Andrea AD, Drané P, De S, Konstantinopoulos PA, Masson JY, Chowdhury D. (2018) DYNLL1 binds to MRE11 to limit DNA end resection in BRCA1-deficient cells. Nature. 563(7732):522-526.

Crossley MP, Song C, Bocek MJ, Choi JH, Kousorous J, Sathirachinda A, Lin C, Brickner JR, Bai G, Lans H, Vermeulen W, Abu-Remaileh M, Cimprich KA. (2023) R-loop-derived cytoplasmic RNA-DNA hybrids activate an immune response. Nature. 613(7942):187-194.

Makharashvili N, Tubbs AT, Yang SH, Wang H, Barton O, Zhou Y, Deshpande RA, Lee JH, Lobrich M, Sleckman BP, Wu X, Paull TT. (204) Catalytic and noncatalytic roles of the CtIP endonuclease in double-strand break end resection Mol Cell. 54(6):1022-1033.

Kim MY, Jung AR, Shin D, Kwon H, Cho HJ, Ha US, Hong SH, Lee JY, Kim SW, Park YH. (2021) Niclosamide exerts anticancer effects through inhibition of the FOXM1-mediated DNA damage response in prostate cancer. Am J Cancer Res. 11(6):2944-2959.
